# Spray Drying of Double-Layer Emulsion Stabilised with an Orange Residue: Effect of Process Parameters and Collection Position

**DOI:** 10.3390/foods14162919

**Published:** 2025-08-21

**Authors:** Mónica Umaña, Esperanza Dalmau, Carmen Rosselló, Valeria Eim, Susana Simal

**Affiliations:** Department of Chemistry, University of the Balearic Islands, Ctra. Valldemossa km 7.5, 07122 Palma de Mallorca, Spain; monica.umana@uib.es (M.U.); esperanza.dalmau@uib.es (E.D.); carmen.rossello@uib.es (C.R.); valeria.eim@uib.es (V.E.)

**Keywords:** spray drying, double-layer emulsion, orange residue, pectin, encapsulation efficiency, microencapsulation, microstructure, response surface methodology, optimisation

## Abstract

This study investigated the impact of spray-drying conditions, specifically inlet air temperature (Tin: 131–159 °C) and feed rate (FR: 4.9–8.4 g/min), on the microencapsulation of oil in a double-layer emulsion stabilised with orange residue flour (ORF) and soy protein. Powders were analysed separately from the drying chamber and the collector, focusing on yield, encapsulation efficiency, moisture, water activity (aw), oil oxidation, colour, and particle size. Chamber powders were more sensitive to Tin, where higher temperatures (155–159 °C) improved yield (up to 47% dry matter (dm)) but also increased oxidation (up to 134% above initial oil). Excessively high FR (8.4 g/min) reduced yield and raised aw (up to 0.39). Collector powders showed more stable yields (average 30 ± 2% dm) but lower encapsulation efficiency (80–86% for chamber vs. 70–77% for collector). Response surface methodology satisfactorily modelled key parameters (R^2^ up to 0.9). Optimisation showed that chamber performance was maximised at 146 °C and 4.9 g/min (predicted yield and aw of 41% and 0.25, respectively), while collector quality improved with slightly higher Tin (150 °C, predicted aw of 0.32). Separately analysing chamber and collector fractions provided novel insights into spray-drying dynamics. These findings highlight ORF as a promising wall material.

## 1. Introduction

Microencapsulation is a widely applied technique used to protect bioactive compounds from undesirable reactions [1]. This process involves coating or entrapping a sensitive core material within one or more biopolymeric wall materials, thereby shielding it from adverse environmental conditions [2]. Among the most used wall materials are polysaccharides, such as maltodextrin, which is valued for its low cost, high water solubility, and good film-forming properties. However, its low affinity for lipophilic compounds often necessitates its combination with proteins or emulsifiers [3,4,5].

In recent years, aligned with sustainability principles and the circular economy, there has been growing interest in replacing conventional encapsulating agents with natural, renewable sources. Agro-food industry residues are increasingly explored as sources of functional polysaccharides and antioxidants [6]. For instance, Burgos-Díaz et al. [7] reported that lupin byproducts (rich in protein and fibre) enhanced the oxidative stability of D-limonene when used as spray-drying wall material. Orange residues, also rich in polysaccharides and antioxidants [8,9], are generated in large amounts during industrial processing, representing 55–60% of fruit weight [10]. They are particularly high in pectins (~22% dry matter) [11,12], polysaccharides mainly composed of galacturonic acid [13], which are valued for their emulsifying and film-forming properties [14,15].

Pectin–protein double-layer emulsions are attracting attention for their ability to form stable electrostatic complexes at pH values between the protein’s isoelectric point and the polysaccharide’s pKa [16,17,18]. In such systems, proteins first adsorb at the oil–water interface, followed by a pectin layer, yielding stable emulsions. In our previous work [13], we developed a double-layer emulsion stabilised with orange residue flour rich in pectin, showing promising emulsifying properties for spray drying. Such double-layer emulsions are precursors to multilayer microcapsules, which can offer enhanced protection, improved storage stability, and controlled release when spray dried [16,19]. In fact, a recent study exploring spray-dried grape products demonstrated that combining protein–polysaccharide wall materials markedly improved antioxidant retention during storage, further underscoring the value of multilayer systems for preserving labile bioactives in complex matrices [20].

Among the various encapsulation techniques, spray drying is the most widely used method in the food industry for oil microencapsulation [3]. This process transforms emulsions into stable powders by atomising them into a stream of hot air, providing protection against oxygen, light, and heat. However, optimal spray-drying conditions are crucial for maintaining the desired physicochemical and functional properties.

In recent years, several studies have employed response surface methodology (RSM) to optimise spray-drying conditions for food emulsions, due to its capacity to model the interactions between critical process variables such as inlet air temperature and feed flow rate. For example, Zahran et al. [1] optimised the microencapsulation of oil by simultaneously adjusting the inlet temperature (140 °C) and the feed flow rate (4.5 mL/min), achieving high encapsulation efficiencies and yields under experimentally validated conditions. Similarly, Cûlina et al. [21] applied RSM to encapsulate sea buckthorn oil using different wall materials (gum arabic, β-cyclodextrin, and their blends). They identified optimal temperature and wall material-to-oil ratios for each formulation. More recently, Dang et al. [22] optimised the spray drying of a W/O/W acerola emulsion, finding optimal conditions at 157 °C inlet and 91 °C outlet temperature, with 91.15% process yield and encapsulation yields of 61.44% for total polyphenols. As stated, spray drying is widely studied to produce microcapsules; however, most research focuses on the powder collected at the end of the process, without distinguishing between the fractions deposited in the drying chamber and those recovered in the cyclone or collector.

Nevertheless, these two fractions undergo distinct drying dynamics and thermal conditions, which can result in significant differences in their physicochemical and functional properties. Only a minimal number of studies, such as Donz et al. [23], have addressed this separation. They analysed powders produced from a high-fat oil-in-water emulsion (50%) collected from different points of an industrial spray dryer, specifically, from the drying chamber, the cyclones (fines 1 and 2), and the final product. They found that the powder collected directly from the chamber had distinct properties compared to the fines. For example, chamber powder showed lower free fat content and better rehydration properties, while fines from cyclones had smaller particles, higher surface fat, and poorer stability when reconstituted.

Despite the extensive research on spray drying for microencapsulation, there is still a lack of studies addressing how the drying history of different collection points (e.g., chamber vs. cyclone collector) influences the physicochemical and functional properties of the resulting powders, particularly when using sustainable, residue-derived wall materials. Understanding these differences is crucial to optimising process conditions for both yield and product quality.

Building on these findings, this study aims to evaluate the effect of spray-drying conditions on the microencapsulation of oil in a double-layer emulsion stabilised with a natural wall material derived from orange residues. The process variables studied were inlet air temperature and feed rate. The emulsion was prepared using pectin-rich orange residue flour combined with proteins to form a protein–polysaccharide complex. In addition, we separately collected and characterised the powders obtained from the drying chamber and the cyclone collector. This distinction, still rarely addressed in the literature, allows for a better understanding of how the different thermal and drying histories of each fraction influence their physicochemical and functional properties. The ultimate goal is to generate knowledge that supports the optimisation of spray-drying processes to improve both yield and product quality when using sustainable wall materials. To this end, response surface methodology was employed to model and optimise the behaviour of each fraction.

## 2. Materials and Methods

### 2.1. Reagents

Monohydrate citric acid, hydrochloric acid (37%), isopropanol (extra pure), and hexane (extra pure) were purchased from Scharlau (Barcelona, Spain), and disodium phosphate (≥99%) and activated aluminium oxide were purchased from Sigma-Aldrich (Madrid, Spain).

### 2.2. Raw Matter

For the preparation of the emulsions, Glucidex^®^ maltodextrin DE 12 (Roquette, Beinheim, France) was used as the primary wall material. Soy protein was supplied by Manufacturas Ceylan S.L. (Valencia, Spain), while sunflower oil was purchased from a local store in Mallorca, Spain. The oil was subjected to a stripping process to purify it and remove antioxidants, such as tocopherols, that were added during industrial production. This procedure is described by Hernández-Sánchez et al. [24]. Briefly, oil purification was performed by vacuum filtration through activated alumina (200 g alumina per 400 g oil) at room temperature. The alumina was preheated at 200 °C for 3 h and cooled before use. The oil was filtered twice using fresh alumina, each time, with each filtration taking approximately 10–15 min. Purified oil was stored frozen in light-protected, airtight containers until use.

The obtainment and characterisation of the orange residue flour (ORF) have been extensively described in our previous study [12]. Briefly, Navelina oranges from a local market (Mallorca, Spain) were submitted to juice extraction. The remaining residue (comprising peel and pulp) was scalded to inactivate endogenous enzymes, freeze-dried (LyoQuest, Telstar, Barcelona, Spain, −50 °C, 30 Pa), ground (ZM 200, Retsch^®^, Haan, Germany), and sieved to obtain particles smaller than 0.18 mm (FIT-0200, Filtra, Barcelona, Spain). In this work, the flour was ground more finely than in our previous study (where a 0.5 mm cut-off was used) to ensure that the spray drying nozzle would not become obstructed.

Based on the characterisation provided in this study [12], the ORF was mainly composed of carbohydrates (over 90 g/100 g dm (dry matter)), with a significant portion being dietary fibre (about 38 g/100 g dm). Within the fibre fraction, pectins were highly abundant (~22 g/100 g dm), as indicated by the high uronic acid content. The pectins in ORF were classified as low-methoxyl pectins (degree of methylation ~41%), which enhances their ability to interact with proteins through electrostatic interactions. Besides pectins, ORF contained small amounts of proteins (~3.6 g/100 g dm), lipids (~1.1 g/100 g dm), and minerals (~3 g/100 g ash). It was also rich in bioactive compounds, with total polyphenols around 16 mg gallic acid equivalent/g dm, and antioxidant activity of ~25 mg Trolox equivalents/g dm (CUPRAC assay).

Additionally, in this work, the ORF was characterised in terms of solubility according to the method described by Pieczykolan et al. [25] with some modifications. To determine solubility, approximately 1 g of powder was dispersed in 100 mL of McIlvaine buffer (pH 3.8) to simulate the conditions of the prepared emulsions. The mixture was stirred for 5 min and then centrifuged. A 25 mL aliquot of the supernatant was dried at 105 °C for 24 h, and the solubility (%) was calculated based on the dry residue relative to the initial sample.

### 2.3. Emulsion Preparation

A double-layer emulsion, consisting of a protein layer and a pectin-rich layer from ORF, was prepared. This process was based on the layer-by-layer technique previously described in our earlier study [12]. However, in this work, some modifications were introduced for technical reasons, including the use of McIlvaine buffer at pH 3.8 instead of the previously used buffer at pH 3.5, and an adjustment in the oil content to improve processability.

The McIlvaine buffer was used, prepared according to McIlvaine [26] by mixing 177.5 mL of 0.2 M disodium phosphate with 322.5 mL of 0.1 M citric acid to obtain a final pH of 3.8. This buffer was selected because it is mild and less aggressive compared to other acidic solutions, while still providing the required acidity for the process. A slightly acidic pH was necessary to ensure that the soy proteins carried a net positive charge, favouring their electrostatic interaction with the negatively charged pectins present in the ORF. Furthermore, pH 3.8 was chosen as it remains above the critical limit of 3.5, which could damage laboratory spray-drying equipment.

The formulation comprised 33.32 g maltodextrin, 4.00 g sunflower oil, 2.38 g ORF, and 0.30 g soy protein, per 100 g of total emulsion, giving 40% (*w*/*w*) total solids, with the remaining 60% (*w*/*w*) consisting of water/buffer. The selected ORF content (2.38 g) provided approximately 0.5 g of pectin (based on its measured pectin content of ~21 g/100 g wet matter), which matches the pectin levels commonly used in previous studies to produce stable bilayer emulsions [16,17,18]. Protein content was set to avoid the bridging flocculation observed at higher concentrations in our earlier study [12]. The oil content, which in our previous work was 6%, was reduced to 4% in this study, since preliminary trials showed that encapsulation efficiency decreased markedly at oil concentrations above 4%. The total solids content was set to 40% based on our previous work and on several studies that have reported improved spray-drying performance at this concentration range [24,27,28,29,30].

Maltodextrin was first dissolved in the McIlvaine buffer (pH 3.8) using half of the total water in the formulation. The ORF was then added, kept under agitation for 2 h, and hydrated overnight. This maltodextrin–ORF dispersion was homogenised with an Ultra-Turrax© (T25 Digital, IKA, Königswinter, Germany) at 16,000 rpm for 8 min to reduce the size of the insoluble ORF particles. Separately, the soy proteins were dispersed in water, stirred and hydrated overnight, after which the pH was adjusted to 3.8 with 4.0 M HCl. A pre-emulsion was then prepared by adding the oil to the protein suspension and homogenising for 10 min at 16,000 rpm. Finally, the pre-emulsion was combined with the maltodextrin–ORF solution and homogenised for an additional 5 min at 16,000 rpm.

Additionally, a control suspension was prepared following the same formulation and procedure as the emulsion, but without the addition of oil. This allowed the evaluation of the particle size distribution of the ORF particles dispersed in the continuous phase.

A fresh emulsion batch (250 g) was prepared immediately before each experimental run to ensure consistent composition and stability prior to spray drying.

### 2.4. Emulsion Characterisation

#### 2.4.1. ζ-Potential

The ζ-potential was measured in the protein pre-emulsion to ensure that it shows a positive charge and, in the emulsion, using a Nano Zetasizer (Nano ZS90, Malvern, UK). Before analysis, the samples were diluted at a 1:500 ratio to minimise multiple scattering effects [31], and the pH of the diluted samples was adjusted back to the original value (3.8). Measurements were performed at 25 °C, and the ζ-potential was calculated from the electrophoretic mobility of the droplets using the Smoluchowski model.

#### 2.4.2. Particle Size and Microstructure of the Initial Emulsions

The particle size distribution (PSD) of the emulsion and the ORF control suspension was determined using a Microtrac MRB equipped with the FlowSync accessory (Verder Scientific, Haan/Düsseldorf, Germany). This instrument measures particle size based on laser diffraction (LD) and dynamic image analysis. For the measurements, approximately 0.2 mL of each sample was introduced into the instrument. In this study, in addition to the volume-based PSD provided by the Microtrac, two key parameters from the instrument were selected: the 50th percentile (d50) as the representative particle diameter and the sphericity as an indicator of particle morphology.

Additionally, the PSD of the emulsions was determined by optical microscopy and image analysis (IA) using an Olympus BX60 microscope (Optical Co., Ltd., Tokyo, Japan) connected to a Moticam 3 camera (Vancouver, Canada), as described previously with some modifications [27]. The emulsions were diluted in distilled water (1:10 *v*/*v*) and immediately observed under a 20× objective. For each sample, a minimum of 10 micrographs were taken, corresponding to at least 4000 droplets analysed. Before image analysis, the micrographs were manually cleaned to remove insoluble ORF particles that appeared in the field of view. Image processing and analysis were performed using ImageJ2 software (version 2.16.0/1.54p). The workflow included the following steps: Convert to Mask, which binarises the image; Fill Holes, which corrects incomplete particle boundaries; and Watershed, which separates touching droplets by creating dividing lines. Finally, the “Analyse Particles” function was applied to automatically detect each droplet, measure its area based on the calibrated scale, and generate a summary of the size distribution. Assuming spherical geometry, droplet diameters and volumes were calculated from the measured areas. From this point onward, LD (laser diffraction) will refer to results obtained using the Microtrac analyser, while IA (image analysis) will denote data derived from optical microscopy combined with image analysis.

This characterisation was performed for each prepared batch of the initial emulsion, and the average PSD, median diameter (d50), and sphericity values are reported.

### 2.5. Spray Drying

The spray-drying process was performed using a Büchi B-290 spray dryer (Buchi, Flawil, Switzerland). The aspiration rate was set to 31.5 m^3^/h (90% of the equipment capacity), while the atomisation airflow was adjusted to 0.83 m^3^/h (corresponding to 45 mm on the rotameter). The outlet temperature (Tout) was monitored continuously during the drying process and is reported as a range in Table 1.

A central composite circumscribed (CCC) design, belonging to the response surface methodology (RSM), was employed to evaluate the effect of two key spray-drying variables: inlet air temperature (Tin) and feed flow rate (FR). The design was centred at 145 °C for Tin and at a FR setting of 20% on the spray dryer, which was experimentally determined to correspond to an actual FR of approximately 6.8 g/min. The step sizes were equivalent to 10 °C for Tin and ±5% in the pump setting for FR. The design comprised a total of 12 experimental runs, including factorial points, axial points, and four replicated centre points. These experiments are presented in Table 1 in the order in which they were conducted. From this point onward, the experiments will be referred to by their corresponding Tin values and feed rates expressed in g/min.

The selected operating ranges were defined based on preliminary experiments, some of which were published in a conference [32]. Some experiments showed that lower inlet temperatures were insufficient for proper drying, while higher flow rates caused unstable atomisation and poor powder recovery. Furthermore, excessively high inlet temperatures were avoided to reduce unnecessary energy consumption and maintain a more sustainable drying process. Additionally, the chosen conditions were consistent with values commonly reported in the literature for similar emulsions [1,16,22,33].

This design was selected because it allows the estimation of linear, interaction, and quadratic effects, which are expected in spray-drying processes where the responses often exhibit non-linear behaviour [1,22]. Moreover, the CCC design provides a reduced, yet efficient number of experimental runs compared to a complete factorial design, while ensuring sufficient information to model. At the end of each drying run, the powder deposited in the drying chamber and the powder recovered from the cyclone collector were collected separately. Each fraction was stored (samples were vacuum-packed in plastic bags and stored in a freezer at −20 °C until analysis) and characterised independently.

#### Powder Yield

The powder collected from the drying chamber and the cyclone collector was weighed separately after each spray-drying run. The moisture content of each powder fraction was then determined, as described later in this section. The yield for each fraction was calculated on a dry matter basis by correcting the collected powder weight for its moisture content and dividing this dry weight by the total solids present in the initial emulsion. Yields were expressed as % dry matter (dm). Finally, the total yield was obtained as the sum of the chamber and collector yields, providing a comprehensive evaluation of the overall process efficiency.

### 2.6. Powder Characterisation

#### 2.6.1. Encapsulation Efficiency

Surface oil content was measured following the Niro Analytical Method A 10a [34] extracting it with ethyl ether. Total oil content was determined using a modified procedure based on Kim et al. [35]. Briefly, the spray-dried powders were reconstituted in distilled water, and oil extraction was performed using 50 mL of a hexane/isopropanol mixture (3:1 *v*/*v*) with agitation for 15 min, followed by centrifugation at 3900 rpm for 15 min. The collected organic phase containing the extracted oil was evaporated. The amount of encapsulated (core) oil was calculated by subtracting the surface oil from the total oil content. Encapsulation efficiency was then expressed as the percentage of oil retained within the microcapsule core relative to the total oil present.

#### 2.6.2. Moisture Content and Water Activity (aw)

The moisture content of each powder was determined as described by the AOAC 934.06 method [36]. The aw was determined at 25 °C using a NOVASINA Thermoconstanter TH200 (Novasina, Lachen, Switzerland).

#### 2.6.3. Oxidation of the Oil

The oxidation level of the oil in the powders was assessed by measuring the specific absorbance (SA) of conjugated dienes at 234 nm. To do this, the emulsion was reconstituted in water (0.5 g in 2 mL of water), and the oil was extracted by adding 50 mL of a hexane–isopropanol mixture (3:1 *v*/*v*). The absorbance of the extracted oil was then recorded at 234 nm, and SA was calculated using Equation (1) [37]. Likewise, the SA of the oil prior to emulsification and spray drying was determined by directly diluting it in hexane–isopropanol (3:1 *v*/*v*) and measuring its absorbance at 234 nm.

(1) $$\mathrm{SA}=\frac{Absorbance\;234\;nm}{Wg}$$

where Wg is the oil mass (g) in 100 mL of the solvent solution analysed.

#### 2.6.4. Microstructure and Size of the Reconstituted Emulsion

The spray-dried emulsions were reconstituted by dispersing 2 g of powder in 3 mL of water at 50 °C and stirring until fully homogenised. The PSD of the reconstituted emulsions was evaluated using both Microtrac analysis (LD) and optical microscopy combined with image analysis (IA), following the same procedures previously described for the initial emulsions. The PSD and d50 obtained from both techniques, as well as the sphericity determined by Microtrac, are reported.

#### 2.6.5. Microstructure, Size, and Colour of the Dry Powder

The powders were examined using scanning electron microscopy (SEM). Samples were mounted on aluminium stubs with double-sided carbon adhesive tape and coated with a thin layer of gold by sputtering. Micrographs were acquired with a HITACHI S-3400N scanning electron microscope (Hitachi, Tokyo, Japan) operated at an accelerating voltage of 15 kV under a vacuum pressure of 40 Pa.

The PSD of the spray-dried powders was measured in dry mode using the Microtrac SYNC analyser (Haan/Düsseldorf, Germany) equipped with the TurboSync dry dispersion accessory. For the powders, number-based distributions were preferred, as large agglomerates can distort results when expressed as volume or weight distributions.

The colour of the powders was determined using a CM-5 colourimeter (Konica Minolta, Tokyo, Japan) with a D65 illuminant, a 2° observer. The CIELab* coordinates, L* for lightness, a* for the red–green axis, and b* for the yellow–blue axis, were obtained.

The browning index (BI) was subsequently calculated from the obtained CIELab coordinates using the standard equation proposed by Buera et al. [38] according to Equations (2) and (3).

(2) $$x=\frac{\left(a^{*}+1.75L^{*}\right)}{\left(5.645L^{*}+a^{*}-3-012b^{*}\right)}$$

(3) $$\mathrm{BI}=100\;(\frac{x-0.31}{0.17})$$

### 2.7. Statistical Analysis

A three-way analysis of variance (ANOVA) was performed to evaluate the effects of inlet temperature (Tin), feed rate (FR), and powder collection position (chamber vs. collector), as well as their two-way and three-way interactions, on each response variable. The analysis was carried out using R Statistical Software (R Core Team, version 4.3.3, December 2024) with RStudio IDE 2025.05.0. When only the main effects were significant, Tukey’s Honestly Significant Difference (HSD) test was applied to compare the levels of the corresponding factor. If significant two-way interactions were detected, Tukey’s test was conducted on the interaction terms to identify which factor combinations differed. Finally, when the three-way interaction was significant, post hoc comparisons were restricted to the levels of this interaction. All results are presented as mean ± standard deviation.

#### 2.7.1. Modelling

The regression coefficients of the response surface models were obtained using a *stepwiselm* function for stepwise multiple regression of Matlab^®^ R2025a (The MathWorks, Natick, MA, USA). This procedure starts from an intercept-only model and iteratively adds or removes candidate terms based on their statistical contribution to explaining the response variable. In our case, the candidate terms included the linear, quadratic, and interaction effects of the two spray-drying variables: Tin and FR. The stepwise algorithm evaluates the inclusion or exclusion of terms by comparing the change in the residual sum of squares (SSE) through an F-test. A *p*-value entrance tolerance of 0.05 was used, while the default exit tolerance was 0.10.

The final equations were second-order polynomial models, typical of response surface methodology (RSM), containing only the terms that meaningfully contributed to describing the variability of each response. This approach balances model parsimony with explanatory power, avoiding overfitting while retaining essential effects. Separate models were generated for each powder collection position (chamber and collector) to account for their distinct drying dynamics. The standard residuals (differences between experimental and calculated values) were obtained with the regression function of Excel version 16.99.2 (25072714) and plotted versus the calculated data to evaluate if they behaved randomly.

#### 2.7.2. Multifactorial Optimisation

To simultaneously optimise multiple characteristics of the obtained powders, a multi-response optimisation approach was implemented based on the Derringer desirability function methodology [39]. Only the responses for which a statistically significant predictive model was obtained were included in the optimisation process. Each selected response was transformed into an individual desirability function, scaled within the interval [0,1], where 0 represents an entirely undesirable response and 1 a fully desirable one. Depending on the target for each response (maximise or minimise), the corresponding transformation function was applied. The overall desirability function D was then computed as the geometric mean of the individual desirabilities. The optimum variables, Tin and FR, were identified to maximise the overall desirability function by using the *fmincon* function of Matlab^®^ 2025a. Separate optimisations were carried out for each collection position (chamber and collector) and a global optimisation combining key parameters of both fractions. The optimisation was initialised from the experimental centre point (145 °C and 6.8 g/min).

Contour plots were generated to visualise the predicted responses across the experimental domain. The Matlab *contour* function was then used to plot isolines for each response, allowing visualisation of trends and interactions between Tin and FR. The optimal conditions obtained were superimposed as a marker on the corresponding contour plots for clarity. After the optimisation, emulsions were spray dried under the selected optimal conditions, and the resulting powders were characterised.

## 3. Results

### 3.1. Orange Residue Flour and Initial Emulsion Characteristics

The chemical composition of ORF has been previously described in the methodology section. Additionally, its solubility at pH 3.8 was measured, yielding a value of 38 ± 2%. The water-soluble fraction of ORF at pH 3.8 (38 ± 2%) is probably composed of pectins, simple sugars, and other soluble dietary fibre components such as arabinans and galactans, together with a small fraction of soluble proteins and phenolic compounds. The remaining insoluble fraction is primarily constituted by cellulose, hemicelluloses with low solubility, and pectins strongly bound to the cell wall matrix. Although pectins are generally considered water-soluble, their dissolution is often slow and incomplete [40]. In composite materials like ORF, this process is further hindered by the structural association of pectins with other cell wall components. The solubility observed in this study is within the upper range reported by Garau et al. [41] for orange peel and pulp dried at moderate temperatures (25–38%) when measured in non-acidified water. Although the values are not directly comparable due to methodological differences, including the use of acidified water in our case, their results support the idea that a large proportion of orange residues remains insoluble under typical aqueous extraction.

From a functional perspective, this partial solubility means that a fraction of ORF remains as suspended particles in the aqueous phase, which can contribute to emulsion stability via Pickering-type stabilisation, as we previously reported for ORF [12] and other partially insoluble plant materials, such as artichoke bracts [27] and mushroom flour [28,42]. In these cases, the presence of insoluble polysaccharides enhanced resistance to droplet coalescence and improved oxidative stability after spray drying.

Regarding the emulsions, the soy protein pre-emulsion at pH 3.8 exhibited a ζ-potential of 6.5 ± 3.0 mV, confirming that at this pH the protein carries a net positive charge (above its isoelectric point). These conditions were, therefore, suitable for the preparation of a double-layer emulsion. After emulsification with ORF, the system showed a ζ-potential of −23.5 ± 0.6 mV, indicating that the negatively charged pectins of the ORF successfully formed the secondary layer, contributing predominantly to the overall surface charge. These results are consistent with Locali et al. [17], who reported that the isoelectric point of soy proteins is close to pH 4.0 and that working below this value promotes electrostatic attraction with negatively charged pectins, facilitating bilayer formation. In line with our findings, Moser et al. [16,18] also observed a shift in ζ-potential from positive values in protein-only systems to negative values after pectin adsorption, confirming the successful deposition of the secondary layer.

### 3.2. Powder Yield

A three-way ANOVA was performed to evaluate the effects of inlet temperature (Tin), feed rate (FR), and powder collection position (chamber or collector) on the yield and the properties of the powder and the reconstituted emulsions. The results are summarised in Table 2. For yield, all main factors were significant (*p* < 0.05), as well as the interactions Tin × Position and FR × Position.

**Figure 1:**
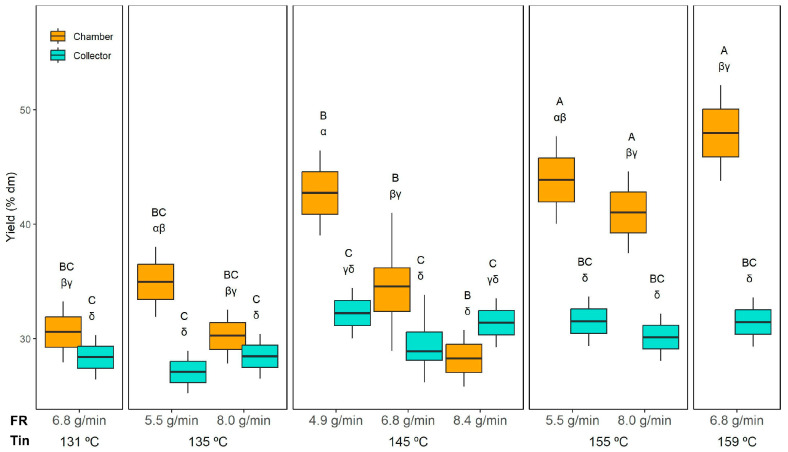
Powder yield obtained at different inlet temperatures (131–159 °C) and feed rates (4.9–8.4 g/min), collected either in the drying chamber or the collector. Different uppercase letters indicate significant differences for Tin × Position, while Greek letters indicate significant differences for the FR × Position interaction (*p* < 0.05).

Figure 1 shows the yield results. Interestingly, the collector yield was practically unaffected by Tin or FR, averaging 30 ± 2% dm across all experiments. The collector yield remained stable because it primarily captures fine, dried particles carried by the exhaust airflow, which are less sensitive to Tin and FR variations. In contrast, the chamber yield varied widely, from 28 ± 2% dm (145 °C and 8.4 g/min) to 48 ± 4% dm (159 °C and 6.8 g/min). When comparing the powder collected from the collector and the drying chamber, significantly higher yields were obtained in the chamber than in the collector, except for the run at 145 °C with 8.4 g/min, where the yield decreased.

Regarding the effect of FR, when comparing samples dried at the same temperature (e.g., 145 °C), a significant decrease in chamber yield was only observed when FR reached the highest value (8.4 g/min). Therefore, 8.4 g/min likely exceeded the dryer’s optimal capacity, introducing too much liquid for the available thermal energy. As a result, the larger droplets formed at this extreme FR required longer drying times, favouring particle stickiness and wall deposition.

Increasing the Tin to 155 °C or 159 °C resulted in significantly higher chamber yields compared to drying at 131 °C or 135 °C. This effect aligns with the outlet temperatures (Tout) recorded under these conditions (Table 1), where higher Tin corresponded to higher Tout, indicating a more efficient drying environment. At elevated Tin, the faster evaporation rate promotes quicker crust formation on droplet surfaces, reducing particle stickiness and wall deposition, thereby enhancing chamber yield. Conversely, at lower Tin (131–135 °C), the lower Tout suggests slower drying kinetics, leaving particles partially wet and prone to adhesion on the chamber walls, which reduces yield.

Overall, these results highlight the importance of optimising Tin and FR to minimise wall deposition in the chamber while maintaining a stable cyclone collection.

Moser et al. [16], who dried a bilayer emulsion containing buriti in a Büchi B-290 at inlet temperatures between 154 °C and 196 °C and feed rates up to 6.8 mL/min, reported total yields ranging from 33% to 54%. In our study, the total yield (calculated as the sum of the powder recovered from the collector and the drying chamber) ranged from 59 ± 2% (135 °C and 8.0 g/min) to 79 ± 6% (159 °C and 6.8 g/min). In both studies, higher inlet temperatures resulted in significantly higher yields. The higher total yields obtained in this study compared to previous works with similar bilayer emulsions may reflect the favourable drying behaviour of the ORF. Similarly, Quintero-Gamero et al. [43], who spray-dried buriti oil emulsions at Tin ranging from 140 °C to 180 °C, achieved total yields between 72% and 96%. As in the present study, higher inlet temperatures favoured higher yields.

### 3.3. Characteristics of the Powders

#### 3.3.1. Encapsulation Efficiency

**Figure 2:**
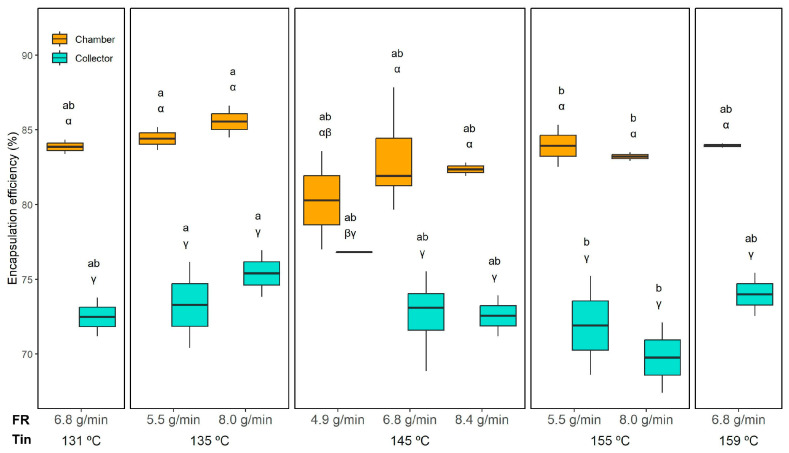
Encapsulation efficiency of the oil into powders obtained at different inlet temperatures (131–159 °C) and feed rates (4.9–8.4 g/min), collected either in the drying chamber or the collector. Different lowercase letters indicate significant differences for Tin, while Greek letters indicate significant differences for the FR × Position interaction (*p* < 0.05).

Encapsulation efficiency (EE) is a key parameter in microencapsulation processes, as it reflects the proportion of oil effectively protected by the wall material. As shown in Table 2, EE was significantly affected by the Tin and the powder collection position, while the interaction FR × Position also had a significant influence (*p* < 0.05). The EE values are shown in Figure 2. As can be seen, among the evaluated factors, collection position had the strongest effect, with the powder collected in the drying chamber exhibiting significantly higher EE. This parameter varied in the chamber from 80.3 ± 3.3% (149 °C and 4.9 g/min) to 86 ± 1.2% (145 °C and 6.8 g/min). For the collector, the lowest encapsulation efficiency was 69.8 ± 2.4% (155 °C and 8.0 g/min), while the highest was 76.8 ± 0.1% (145 °C and 4.9 g/min). Although Tin had a statistically significant effect, it was less pronounced than the influence of the collection position. Nevertheless, a slight decrease in EE was observed at 155 °C compared to 135 °C in both the chamber and collector, regardless of the feed rate. This reduction may be attributed to increased thermal stress at higher temperatures, which can promote partial coalescence or surface oil formation, thereby lowering encapsulation efficiency.

The reported values are similar but slightly lower than those achieved in the investigation of Moser et al. [16], who also dried double-layer emulsions combining proteins and polysaccharides (79–90%). They expressed that probably the bilayer formed at the oil droplet surface contributed to the high encapsulation efficiency. Our results are also comparable to those reported for other emulsions containing vegetable oils spray dried at the laboratory scale. For instance, sea buckthorn berry oil emulsions processed in a Labplant SD-06 at 120–180 °C achieved EE values ranging from 73 to 93%, with higher Tin and the use of β-cyclodextrin improving retention [21]. In contrast, retinoic acid emulsions spray dried in a Büchi B-290 at 115–150 °C showed much lower EE (27–65%) due to the extreme thermal sensitivity of the compound and the less stable emulsions [44]. They observed that intermediate Tin (~130 °C) maximised encapsulation efficiency, while higher Tin (150 °C) led to a decline due to thermal stress and weaker wall integrity.

The higher EE observed in the chamber powder compared to the collector is in general agreement with the observations of Donz et al. [23]. In this study whey–palm oil emulsions with a very high fat content (50 g/100 g) were spray dried at an industrial scale, and powders were collected at different stages of the process: directly from the drying chamber, from the first and second cyclones, and as the final mixed product. They observed that the free surface fat (meaning a low encapsulation efficiency) content increased significantly in the powders processed through the cyclones, with the second cyclone showing the highest free fat levels (around 37%) compared to only ~8% in the final powder and ~3% in the chamber powder without fines. This accumulation of surface fat was attributed to mechanical stress and particle selection in the cyclones, which either released fat droplets from the powder matrix or preferentially carried lighter fat-rich particles.

#### 3.3.2. Moisture Content and Water Activity

**Figure 3:**
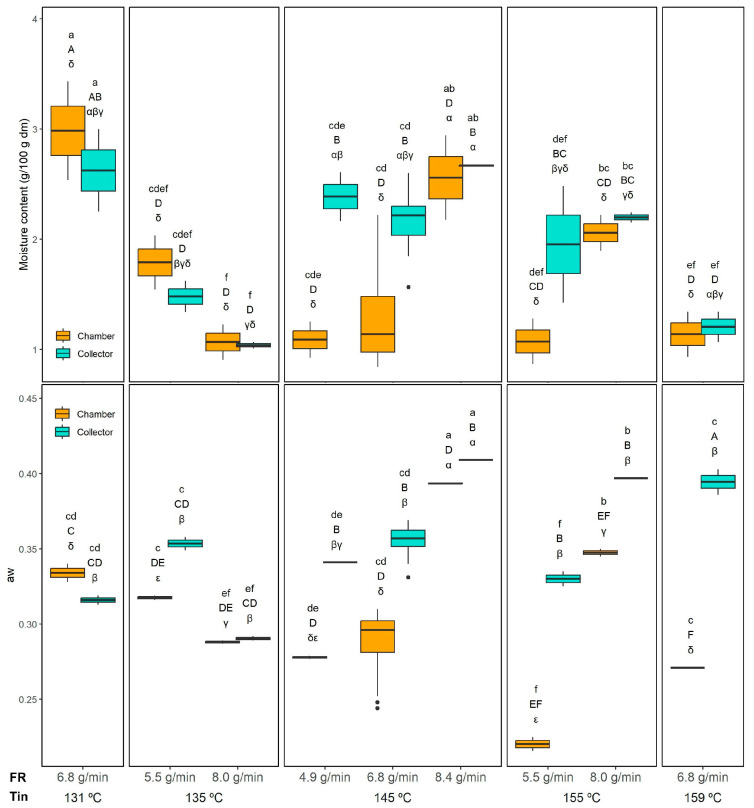
Moisture content and aw of the powders obtained at different inlet temperatures (131–159 °C) and feed rates (4.9 °C and 8.4 g/min), collected either in the drying chamber or the collector. Different lowercase letters indicate significant differences for the Tin × FR interaction, uppercase letters for Tin × Position, and Greek letters for the FR × Position interaction (*p* < 0.05).

Regarding the moisture content (MC), all the evaluated factors (Tin, FR, and powder collection position) showed a significant effect (*p* < 0.05), as well as all two-way interactions. The MC and the aw results are shown in Figure 3. In contrast, the three-way interaction Tin × FR × Position was not significant. The MC ranged from 1.04 ± 0.03 (155 °C 8.0 g/min) to 2.67 ± 0.01 g/100 g dm (145 °C and 8.4 g/min) in the collector and from 0.99 ± 0.15 (145 °C and 6.8 g/min) to 2.98 ± 0.21 g/100 g dm (131 °C and 6.8 g/min) in the chamber. Significant differences between chamber and collector powders were observed only when drying at 145 °C with feed rates of 4.9 and 6.8 g/min, where the chamber powder presented a lower MC, likely due to its longer exposure to higher temperatures inside the drying chamber. At a constant Tin of 145 °C, the MC of the chamber increased when the FR was raised from 4.9 and 6.8 g/min to 8.4 g/min, probably because this higher feed rate exceeded the drying capacity, resulting in insufficient moisture removal. Interestingly, this FR effect was not observed in the collector.

Regarding the Tin effect, when comparing chamber samples dried at the same FR (6.8 g/min), a significant decrease in the MC of the chamber was observed when increasing Tin from 131 °C to 145 °C and 159 °C, which is probably relates to the higher Tout (Table 1) observed in this conditions (145 and 159 compared to 131 °C). A similar trend was also evident in the collector powders, where the MC at 131 °C was significantly higher than at 159 °C, confirming that higher Tin enhances moisture removal efficiency. These results align with the drying dynamics in spray drying. Higher Tin accelerates water evaporation and promotes the rapid formation of a dry outer layer, which reduces overall moisture content. Conversely, excessively high feed rates increase the droplet size and shorten the effective residence time, limiting moisture removal and resulting in higher residual MC.

Comparable MC values have been reported for other emulsions spray dried at the laboratory scale. For instance, sea buckthorn berry oil powders processed at 120–180 °C showed MC values ranging from 0.23 to 3.7%, also decreasing with higher Tin [21]. In contrast, Moser et al. [16] obtained much lower MC values (0.4–1.0%) for bilayer buriti oil emulsions dried at 154–196 °C, likely due to the higher Tin and the use of partial vacuum. Nevertheless, they observed a similar trend with FR, where increasing the feed rate resulted in higher residual MC, although in their case, Tin did not show a clear effect.

Regarding the aw (Figure 3), all the evaluated factors (Tin, FR, and collection position) as well as their two-way interactions were significant (*p* < 0.05), while the three-way interaction Tin × FR × Position was not. The lowest aw in the collector was observed at 135 °C and 8.0 g/min (0.29 ± 0.01), while the highest aw was obtained at 145 °C and 8.4 g/min (0.41 ± 0.01). In the chamber, the lowest aw was recorded at 155 °C and 5.5 g/min (0.22 ± 0.01), and the highest at 145 °C and 8.4 g/min (0.39 ± 0.01). The effect of collection position was not very consistent. Overall, higher aw values were generally observed in the collector compared to the chamber, consistent with the shorter residence time of particles collected in the cyclone. However, this trend was not consistent under all conditions. For instance, at the lowest drying temperatures (131 °C and 135 °C), the chamber and collector exhibited similar aw values, likely because the lower thermal input in the chamber was insufficient to evaporate free water as effectively as at higher temperatures.

The effect of FR was clearer. At 145 °C, the highest FR (8.4 g/min) produced a significantly higher aw than the lower FR conditions (4.9 and 6.8 g/min), both in the chamber and in the collector, which is in agreement with the MC results.

When maintaining the FR constant at 6.8 g/min, increasing Tin from 131 °C to 159 °C significantly reduced the aw in the chamber. In contrast, this trend was not observed in the collector, where aw increased at 159 °C. There is a lack of direct correlation between MC and aw particularly evident in that experiment (159 °C and 6.8 g/min), where the collector powder showed the highest aw despite not having a high MC. The higher aw in this powder, despite a moderate MC, can be explained by the faster surface drying of maltodextrin-based particles at high Tin (and high Tout, Table 1), which reduces total moisture but leaves a larger fraction of the residual water accessible on the particle surface, especially in the finer particles collected in the collector.

Comparable aw values have been reported for other spray-dried emulsions. Čulina et al. [21] observed aw values ranging from 0.22 to 0.35, with slightly lower values at higher Tin. Moser et al. [16], drying bilayer emulsions of buriti oil, obtained even lower aw values (0.18–0.27) due to the higher Tin and the use of partial vacuum. Similarly, Gonçalves et al. [44] reported aw values in the range of 0.20–0.30 for bilayer buriti oil emulsions, where FR had a more pronounced effect than Tin. Despite the differences in absolute values, likely due to the specific wall material composition, all studies confirm that higher FR tends to increase aw. In contrast, the effect of Tin is weaker or only evident at lower FR.

#### 3.3.3. Oxidation of the Encapsulated Oil

The oxidation level of the initial and the encapsulated oil was evaluated by measuring the specific absorbance of conjugated dienes (SA at 234 nm), which are primary oxidation products of unsaturated fatty acids [45]. This parameter is critical, as it reflects both the oxidative stability of the final product and the protective capacity of the wall materials during the spray drying process.

As shown in Table 2, Tin and FR significantly affected oil oxidation, while the collection position alone did not exhibit a strong effect. However, all two- and three-way interactions were also significant (*p* < 0.05), indicating a complex relationship between drying conditions and oxidative stability.

**Figure 4:**
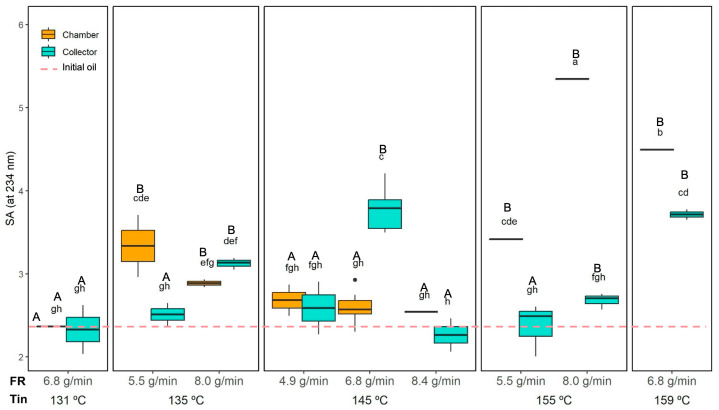
Specific absorbance (SA) of conjugated dienes (234 nm) of the oil before emulsification and spray drying (initial oil) and the oil encapsulated into the powders obtained at different inlet temperatures (131–159 °C) and feed rates (4.9–8.4 g/min), collected either in the drying chamber or the collector. Different lowercase letters indicate significant differences for the Tin × FR × Position interaction, and different uppercase letters indicate significant differences between each sample and the initial oil (*p* < 0.05).

The SA results are presented in Figure 4. The initial oil, before emulsion preparation and spray drying, had a baseline SA of 2.3 ± 0.1. Most of the dried powders exhibited values not significantly different from those of the initial oil, indicating that the process preserved the oil’s integrity. However, some conditions led to significantly higher oxidation levels. In the collector, samples dried at 135 °C and 8.0 g-min, 145 °C and 6.8 g/min, 155 °C and 8.0 g/min, and 159 °C and 6.8 g/min exhibited significantly higher SA than the baseline oil. In the chamber, all powders dried at 135 °C and 155 °C, as well as those at 159 °C (regardless of FR), also showed significantly higher oxidation.

Across all conditions, SA ranged from 2.3 ± 0.2 (145 °C and 8.4 g-min) to 3.8 ± 0.3 (145 °C and 6.8 g/min) in the collector, and from 2.4 ± 0.1 (131 °C and 6.8 g/min) to 5.4 ± 0.1 (155 °C and 8.0 g/min) in the chamber. Significantly higher SA values (*p* < 0.05) were observed in the chamber compared to the collector when drying at 135 °C and 5.5 g/min, at 155 °C (regardless of FR), and 159 °C. This suggests that the longer residence time and greater thermal exposure in the drying chamber promoted oil oxidation compared to the collector. Nevertheless, the higher encapsulation efficiency (EE) of chamber powders may have provided a more effective oxygen barrier, partially offsetting thermal degradation.

In some cases, increasing FR led to higher oxidation (e.g., at 155 °C, chamber powders showed higher SA at 8.0 g/min compared to 5.5 g/min), while in other cases, FR had no significant impact or even reduced oxidation. Conversely, the Tin effect was more consistent: the highest Tin (159 °C) produced significantly higher oxidation in both the chamber and collector compared to the lowest Tin (131 °C) at the same FR. The Tin effect was more pronounced in the chamber where the highest SA values were observed at the highest temperatures (155 and 159 °C; in these conditions, Tout was close to or even higher than 100 °C; see Table 1).

Oxidation patterns varied with collection position. In the chamber, SA values increased steadily with Tin, as longer residence time and higher temperatures promoted cumulative thermal degradation. In contrast, collector powders, with lower encapsulation efficiency and more exposed surface oil, were more sensitive to oxygen. Oxidation peaked at 145 °C and 6.8 g/min because this condition represented the least favourable balance between surface oil protection and oxidative exposure. At this intermediate feed rate, droplet size and moisture content during drying were such that crust formation was slower than at 4.9 g/min, leaving more unprotected oil exposed to oxygen, while the residence time was still sufficient for oxidation to occur. In contrast, at the same temperature but with a lower feed rate (4.9 g/min), smaller droplets and faster crust formation reduced oxygen access, lowering oxidation. Similarly, at a higher feed rate (8.4 g/min), although crust formation was also slower, the shorter residence time in the chamber limited oxygen contact, resulting in lower oxidation. At 155 °C, faster crust formation further restricted oxygen diffusion, slightly reducing oxidation, whereas at 159 °C, extreme thermal stress once again promoted oil degradation despite crust presence.

Similar observations were reported by Javed et al. [46], who spray dried designer egg powder and found that higher inlet temperatures (≥180 °C) significantly increased peroxide values and accelerated PUFA degradation, highlighting the thermal sensitivity of lipids. Likewise, Huang et al. [47] showed that higher Tin (110–123 °C) and lower encapsulation efficiency in tilapia oil powders led to increased hydroperoxide formation. Although different matrices and markers were used (peroxide values or hydroperoxides vs. SA of conjugated dienes), both studies confirm that elevated inlet temperatures promote lipid oxidation.

#### 3.3.4. Size and Microstructure of the Reconstituted Emulsions

**Figure 5:**
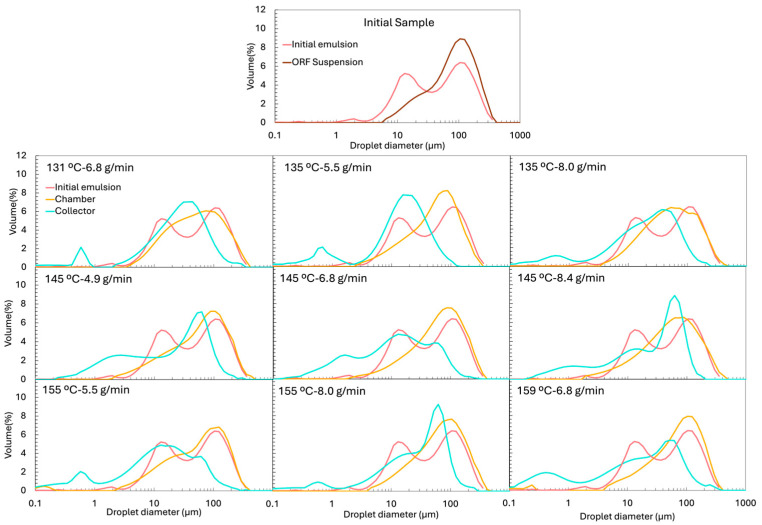
Particle size distribution (PSD) obtained by laser diffraction and dynamic image analysis (LD) of the initial emulsion (before spray drying), the ORF suspension (oil-free), and the reconstituted emulsions obtained from powders produced at different inlet temperatures (131–159 °C) and feed rates (4.9–8.4 g/min), collected either from the drying chamber or the collector. For comparison, the PSD of the initial emulsion was included in all charts to facilitate interpretation.

The particle size distribution (PSD) of the initial emulsion (before spray drying) is presented in Figure 5 and exhibits a clear bimodal profile. The first peak, around 10 µm, corresponds to the oil droplets. In contrast, the second, broader peak (~79 µm) originates from the insoluble particles of ORF, which is only partially soluble (measured solubility was 38 ± 2%). The PSD of the oil-free ORF suspension, also included in Figure 5, confirms its contribution to the large-size population of the initial emulsion.

**Figure 6:**
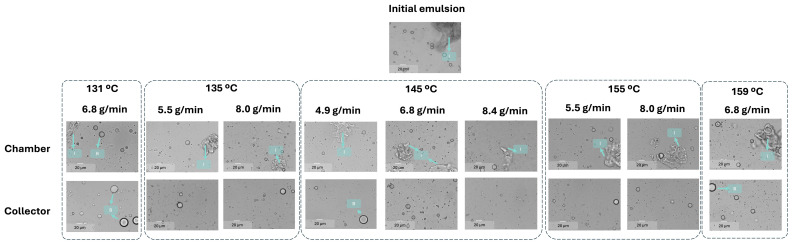
Microphotograph of the initial emulsion (before spray drying) and the reconstituted emulsions obtained from powders produced at different inlet temperatures (131–159 °C) and feed rates (4.9–8.4 g/min), collected either from the drying chamber or the collector. Scale bar represents 20 µm. “I” stands for irregularly shaped particles and “B” for big droplets.

**Figure 7:**
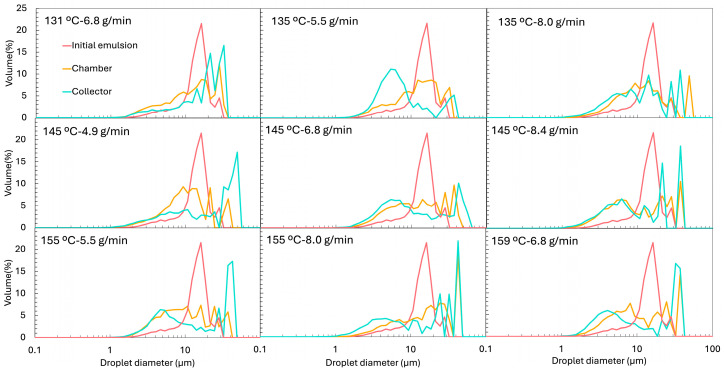
Particle size distribution (PSD) obtained by optical microscopy and image analysis (IA) of the initial emulsion (before spray drying) and the reconstituted emulsions obtained from powders produced at different inlet temperatures (131–159 °C) and feed rates (4.9–8.4 g/min), collected either from the drying chamber or the collector. For comparison, the PSD of the initial emulsion was included in all charts to facilitate interpretation.

This compositional complexity complicates the interpretation of the reconstituted emulsions, since the insoluble ORF particles remain present after drying and reconstitution. To distinguish between droplets and insoluble particles, two complementary techniques were used: (i) laser diffraction and dynamic image analysis (LD), which detects all dispersed elements (droplets + fibres) (Figure 5), and (ii) optical microscopy coupled with image analysis (IA), where insoluble ORF particles were visually excluded, so only oil droplets were analysed (Figure 6 for the micrograph and Figure 7 for the PSD). From the LD PSDs in Figure 5, marked differences were observed between emulsions reconstituted from chamber and collector powders. Collector-derived emulsions showed an additional population of very fine droplets/particles (<1 µm), absent in chamber emulsions. This likely originates from the atomisation step, where intense shear and turbulence can generate submicron droplets that, once dried, are more prone to being carried to the collector. As described by Höhne and Gaukel [48], the atomisation step itself can lead to oil droplet breakup and consequently alter the oil droplet size distribution before the drying stage. A similar effect was reported by Peng et al. [49], who observed a ~65% reduction in droplet size after spray drying, which they attributed to atomiser speed and nozzle size.

Thus, the formation of finer droplets during atomisation may result in a broader PSD in the reconstituted emulsion compared to the original feed. Conversely, the large-size peak associated with insoluble ORF particles was much more prominent in chamber samples but was nearly absent in the collector, suggesting that the heavier insoluble particles were retained in the drying chamber rather than transported to the cyclone. It is possible that, in the chamber powders, the very large ORF particles dominate the volume-weighted PSD, making the few smaller droplets (<1 µm) undetectable.

**Figure 8:**
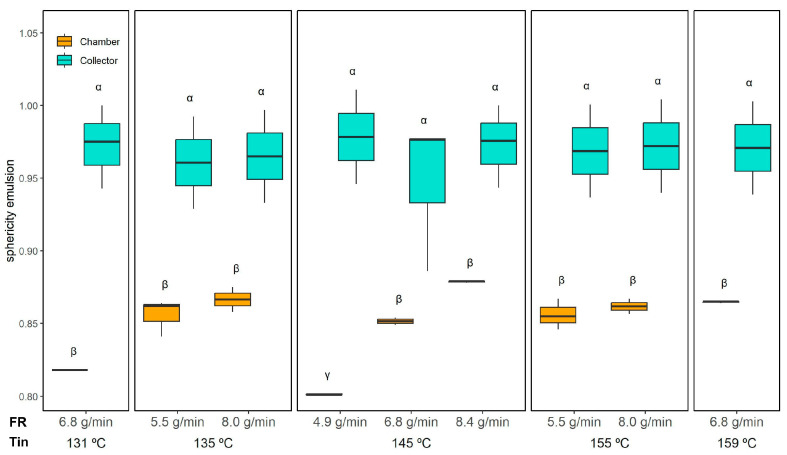
Sphericity of the reconstituted emulsions obtained from powders produced at different inlet temperatures (131–159 °C) and feed rates (4.9–8.4 g/min), collected either from the drying chamber or the collector. Different Greek letters indicate significant differences for FR × Position interaction.

These compositional differences are corroborated by the micrographs in Figure 6, where chamber-derived emulsions exhibit a higher prevalence of irregular particles (“I”), which are attributed to the insoluble fraction of the ORF. Quantification of droplet sphericity (Figure 8) supports this observation: droplets in reconstituted emulsions from collector powder maintained high sphericity (0.97 ± 0.02, unaffected by drying conditions), whereas those from the chamber powder had lower sphericity varying from 0.80 ± 0.01 (145 °C and 4.9 g/min) to 0.88 ± 0.01 (145 °C and 8.4 g/min), similar to the initial emulsion (0.85 ± 0.01), indicating a greater presence of irregular ORF particles.

**Figure 9:**
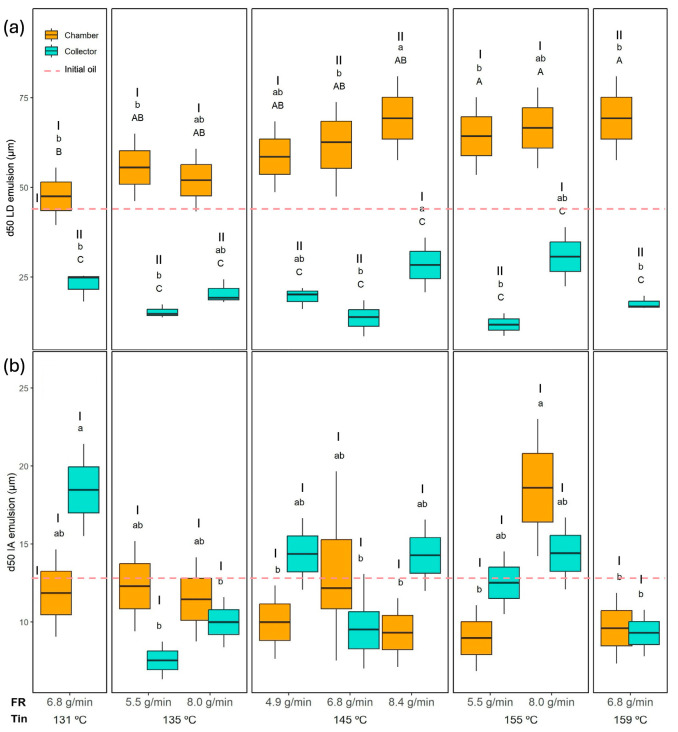
Median size (d50) of the PSD obtained by the LD (**a**) and IA (**b**) of the initial and the reconstituted emulsions obtained from powders produced at different inlet temperatures (131–159 °C) and feed rates (4.9–8.4 g/min), collected either from the drying chamber or the collector. For (**a**), different lowercase letters indicate significant differences for FR and uppercase letters for the Tin × Position interaction; for (**b**), different lowercase letters indicate significant differences for the Tin x FR × Position interaction (*p* < 0.05). For both (**a**) and (**b**), different Roman numbers indicate significant differences with the initial emulsion.

When examining only the droplets via IA (Figure 7), the PSD profiles of reconstituted emulsions from chamber and collector powders became similar. This was also evident when comparing the d50 values of the PSD. These values are shown in Figure 9, presenting the d50 obtained by LD (a) and by IA (b). The d50 of the reconstituted emulsions obtained by IA (Figure 9b) was very similar between the chamber and the collector powders. The collector showed values ranging from 7.5 ± 1.2 µm to 18.5 ± 3.0 µm, while the chamber ranged from 9.3 ± 2.3 µm to 18.6 ± 4.4 µm, without any clear systematic difference. However, when using LD, which includes all dispersed particles, a clear difference between positions emerged. The chamber samples exhibited much higher d50 values (47.5–69.3 µm) compared to those of the collector (11.6–30.7 µm). This disparity reflects the contribution of larger and irregular ORF particles retained in the chamber, which were excluded from the image-based measurement.

It is noteworthy that the chamber powders also exhibited higher encapsulation efficiency as previously described (80–86% vs. 70–77% in the collector), which is consistent with their higher retention of ORF insoluble particles. This higher retention of ORF insoluble particles in the chamber powders can be explained by their relatively high density and larger size, which reduces their likelihood of being entrained in the cyclone airflow and collected in the cyclone vessel. As a result, these particles remain predominantly in the chamber fraction. Their presence contributes to a more continuous and complete wall matrix around the oil droplets, improving encapsulation efficiency. Furthermore, ORF particles are rich in bioactive compounds with antioxidant activity (as described in the methodology regarding ORF composition), which may enhance oxidative stability by limiting oxygen access to the encapsulated oil. This could explain why, despite being exposed to harsher thermal conditions, the oil in the chamber powders showed similar or even lower oxidation levels compared to the collector powders in some conditions (Figure 4, e.g., at 131 °C and 145 °C). Moreover, the chamber powders generally have larger particle sizes compared to the collector fraction (as discussed in Section 3.3.5); it is the combined effect of particle composition and structure that appears to play a dominant role in the improved encapsulation efficiency and oxidative stability observed in the chamber samples.

The initial emulsion, analysed by IA, showed a d50 of 12.7 ± 2.7 µm (Figure 9b), which did not differ significantly from the d50 values of the reconstituted emulsions, whether from the chamber or the collector. However, relying solely on d50 is somewhat limiting, as it only reflects the median and does not capture the full distribution of droplet sizes. For this reason, we also presented the full PSD curves (Figure 7). These curves reveal that the initial emulsion exhibited a much narrower, more monomodal distribution, while the reconstituted emulsions developed broader, bimodal-like distributions, with additional populations shifted both to the left (smaller droplets) and right (larger droplets). This suggests that, during drying and reconstitution, droplet rearrangements and partial destabilisation occur, even if the median size remained unchanged.

Moreover, Figure 6 reveals that the reconstituted emulsions, particularly those from the collector, exhibited some large droplets (marked as “B”), with sizes exceeding 20 µm. Interestingly, the initial emulsion, when analysed by LD, showed an intermediate d50 value (43.9 ± 5.5 µm), positioned between the reconstituted emulsions from the chamber and the collector (Figure 9a).

When comparing the different experiments, no clear or consistent trend was observed regarding the effect of Tin or FR on the droplet size (d50) obtained by IA. Laser diffraction provides complementary information to image analysis, as optical microscopy is limited in detecting very small particles (<0.2 µm) [50], whereas laser diffraction can cover a much broader size range, from approximately 0.01 to 4000 µm [51]. In the collector samples, where microscopy and sphericity results confirmed that most dispersed particles were indeed droplets, the LD results are reliable (Figure 9a). In this case, the highest feed rates (e.g., 145 °C and 8.4 g/min and 155 °C and 8.0 g/min showed d50 of 30.7 ± 8.3 µm and 28.3 ± 7.6 µm, respectively) tended to produce larger d50 values. This trend was also evident in the PSD shown in Figure 5, where the peaks corresponding to larger droplets become more pronounced in these samples, while the peaks associated with smaller droplets diminish. This can be explained by two combined effects: a higher FR reduced atomisation efficiency, producing larger initial spray droplets, and simultaneously lowered the outlet temperature, slowing the drying kinetics, and the longer drying time allowed more droplet collision and coalescence before the particle structure was locked. These observations are in agreement with the results reported by Höhne and Gaukel [48], who spray dried a model oil-in-water emulsion stabilised with modified starch under controlled conditions, systematically varying inlet (180–220 °C) and outlet temperatures (65–85 °C). They observed that higher outlet and inlet temperatures, which increased the drying rate, led to smaller oil droplets in the reconstituted powders. The explanation was that faster drying caused earlier skin formation on the droplet surface, reducing the time for collisions and coalescence during the drying step.

#### 3.3.5. Size, Morphology, and Colour of the Powders

**Figure 10:**
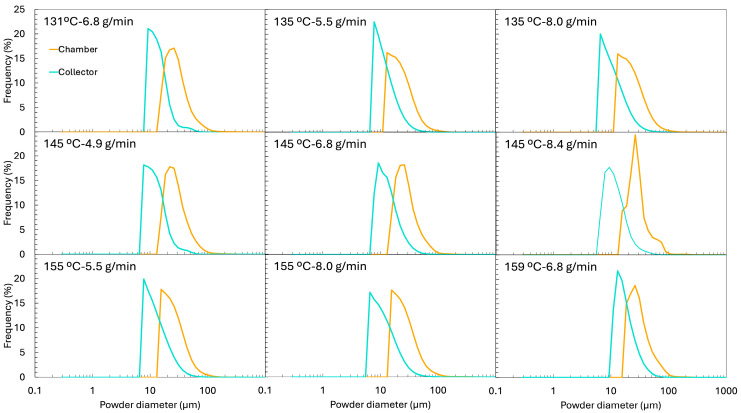
Particle size distribution of the powders obtained at different inlet temperatures (131–159 °C) and feed rates (4.9–8.4 g/min), collected either in the drying chamber or the collector.

**Figure 11:**
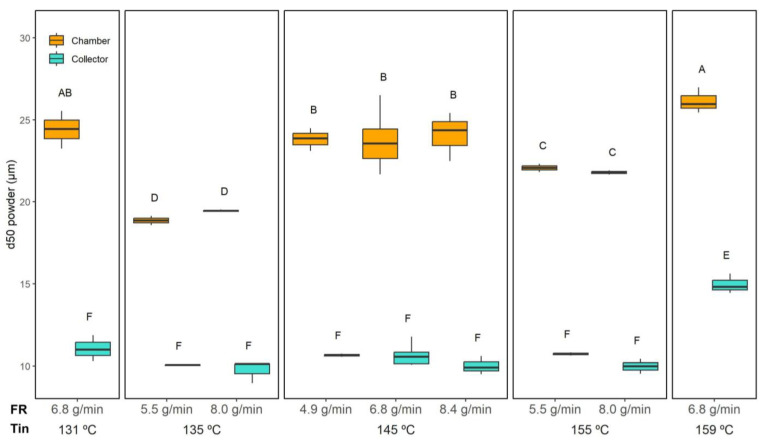
Median of the particle size distribution (d50) of the powders obtained at different inlet temperatures (131–159 °C) and feed rates (4.9–8.4 g/min), collected either in the drying chamber or the collector. Different uppercase letters indicate significant differences for the Tin × Position interaction (*p* < 0.05).

**Figure 12:**
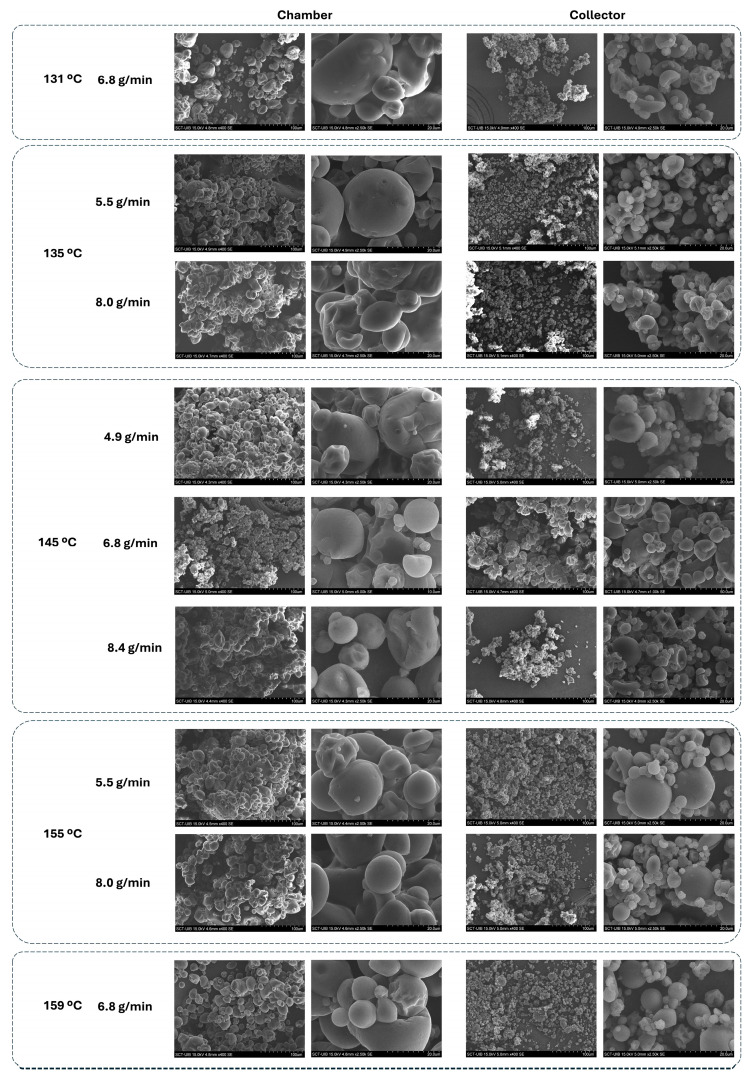
Scanning electron microscopy (SEM) micrographs of powders produced at different inlet temperatures (131–159 °C) and feed rates (4.9–8.4 g/min), collected either from the drying chamber or the collector.

The PSD of the dried powders is shown in Figure 10, while the median particle size (d50) derived from these distributions is presented in Figure 11. Representative SEM micrographs of the microcapsules are provided in Figure 12, allowing a detailed visualisation of their morphology. In this study, a number-based particle size distribution was used instead of a volume-weighted one. Maltodextrin-based powders tend to form mild agglomerates, which strongly bias volume-weighted metrics, as even a small fraction of large particles disproportionately increases the mean diameter. A number distribution is less affected by these agglomerates and better represents the dominant population of individual microcapsules, providing a more realistic comparison between drying conditions.

Across all figures, a consistent trend is evident: powders collected in the chamber displayed substantially larger particle sizes compared to those recovered from the collector.

According to the three-way ANOVA (Table 2), both the collection position and the Tin significantly affected d50 (*p* < 0.05), as did their interaction, whereas FR alone showed no significant effect. In the collector, the PSD was highly uniform, with d50 values remaining remarkably consistent for most conditions (10.4 ± 0.5 µm). The only exception was observed at the highest drying temperature tested (159 °C and 6.8 g/min), where d50 increased to 15.0 ± 0.6 µm.

Both Moser et al. [16] and Gonçalves et al. [44] reported trends that align with this effect of Tin observed in the collector. In Moser et al.’s [16] study, spray-drying buriti oil bilayer emulsions at inlet temperatures between 154 and 196 °C and feed rates from 1.2 to 6.8 mL/min resulted in particle sizes ranging from 17.9 to 32.8 µm (volume-weighted mean diameter). They observed that increasing Tin and FR consistently produced larger particles due to faster crust formation and particle expansion. Similarly, Gonçalves et al. [44], working with retinoic-acid-loaded emulsions encapsulated with different biopolymers, showed that powders dried at higher temperatures (130–150 °C) presented larger d50 values (volume-weighted mean diameter up to ~13 µm with modified chitosan), while milder conditions (115 °C) produced smaller, more uniform particles (~1.7 µm).

In contrast, powders collected from the chamber exhibited greater variability. The largest d50 values were found at the extreme drying conditions, namely, the lowest (131 °C) and highest (159 °C) Tin, both yielding around 25.2 ± 1.2 µm. Intermediate Tin values (135 and 155 °C) resulted in comparatively smaller particles collected in the chamber (20.5 ± 1.6 µm). This pattern suggests that at very low Tin, incomplete atomisation and early deposition promoted larger agglomerates, while at very high Tin, rapid crust formation around partially dried droplets may have led to hollow, bulkier particles. Conversely, mid-range temperatures facilitated more controlled droplet shrinkage, yielding finer particles.

The SEM micrographs (Figure 12) revealed that the powders were generally spherical with smooth surfaces, and no visible fibres from the ORF were detected externally. This suggests that the ORF components were successfully embedded within the microcapsule matrix, likely contributing to crust formation rather than remaining as free particles. The larger sizes in the chamber powders could be linked to their higher encapsulation efficiency, as the additional ORF material retained in the chamber may have thickened the wall structure and promoted particle growth.

**Figure 13:**
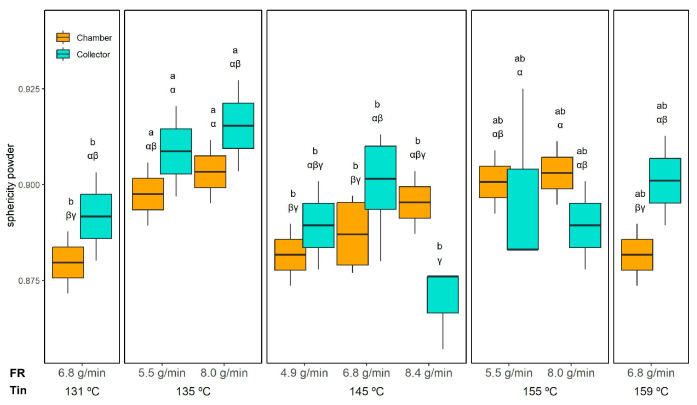
Sphericity of the powders obtained at different inlet temperatures (131–159 °C) and feed rates (4.9–8.4 g/min), collected either in the drying chamber or the collector. Different lowercase letters indicate significant differences for Tin and different Greek letters for the FR × Position interaction (*p* < 0.05).

Sphericity measurements (Figure 13) confirmed that all powders maintained a relatively high degree of roundness, with only subtle differences across conditions. The three-way ANOVA indicated significant effects of Tin, collection position, and the FR × Position interaction; however, these effects were minor. Overall, sphericity ranged from 0.88 ± 0.01 (collector, 145 °C and 8.4 g/min) to 0.91 ± 0.01 (collector, 135 °C/8.0 g/min), indicating well-formed spherical microcapsules across all conditions.

**Figure 14:**
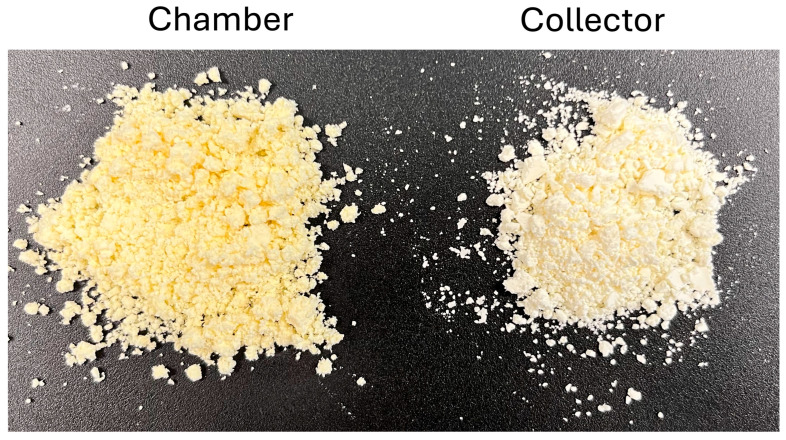
Picture of the powder obtained at 145 °C and 6.8 g/min collected in the drying chamber or the collector.

**Figure 15:**
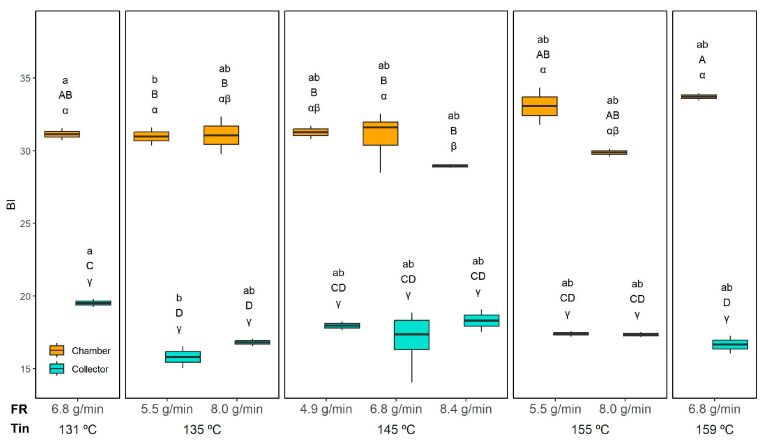
Browning index (BI) of the powders obtained at different inlet temperatures (131–159 °C) and feed rates (4.9–8.4 g/min), collected either in the drying chamber or the collector. Different lowercase letters indicate significant differences for the Tin × FR interaction, different uppercase letters for the Tin × Position interaction, and different Greek letters for the FR × Position interaction (*p* < 0.05).

The colour of the powders was evaluated both visually and instrumentally. Since no major visual differences were observed across drying conditions, Figure 14 presents a representative example of the powders from the collector and chamber at the central drying condition (145 °C and 6.8 g/min). The chamber powder appears darker and more intensely yellow, with a slightly coarser texture and more irregularly shaped agglomerates. In contrast, the collector powder looks lighter, almost pale yellow to off-white, and seems finer with a more uniform, less compact structure. To quantify these observations, the CIELab* coordinates were determined, and from them, the Browning index (BI) was calculated (Equations (2) and (3)) (Figure 15).

The BI values (Figure 15) were significantly influenced by the interaction of all studied variables (Tin, FR, and position). As expected, the collection position had the strongest effect, with chamber powders consistently exhibiting higher BI values than those collected in the collector. In the chamber, the BI ranged from 29.0 ± 0.1 (145 °C and 8.4 g/min) to 33.7 ± 0.3 (159 °C and 6.8 g/min), whereas in the collector, it ranged from 15.8 ± 0.7 (135 °C and 5.5 g/min) to 19.5 ± 0.3 (131 °C and 6.8 g/min).

This difference can be explained by three main factors. First, the powders collected in the chamber contain a higher proportion of orange residue flour (ORF), which inherently imparts a stronger colour due to its natural pigments. Second, ORF is rich in sugars that can enhance browning reactions, further increasing the BI. Third, the longer residence time of particles in the chamber exposes them to prolonged heat, favouring Maillard-type reactions.

Within the chamber powders, the FR showed only a minor effect: at the highest FR (8.4 g/min), the BI slightly decreased, likely because faster drying reduced the residence time and thermal load and also because a higher FR can reduce the Tout. This is in agreement with Moser et al.’s [16] observations; they reported that buriti oil bilayer emulsions spray dried with varying Tin (154–196 °C) and FR (1.2–6.8 mL/min) showed pigment degradation and colour changes with higher Tin, whereas a higher FR mitigated these effects by reducing exposure time. A similar trend was reported for spray-dried spinach juice, with the BI increasing significantly at higher inlet temperatures (160–200 °C) but decreasing when the outlet temperature was reduced from 100 to 80 °C by increasing the feed rate [52,53,54]. In any case, this effect was negligible compared to the strong influence of collection position in our study. In the collector, no clear trend was observed for either the FR or Tin, with BI values remaining relatively uniform.

### 3.4. Modelling

Response surface methodology (RSM) was applied to evaluate the effects of spray-drying process variables (Tin and FR) on the characteristics of the powders and the reconstituted emulsions. Experimental data were fitted to a second-order polynomial model, and the regression coefficients for each response are shown in Table 3. Only responses for which the proposed model was statistically significant (*p* < 0.05) are reported. Model adequacy was evaluated through R^2^ and adjusted R^2^ values, and only models with acceptable explanatory power (R^2^ > 0.5) were considered.

For the chamber fraction, the model was statistically significant and exhibited acceptable explanatory power for yield, aw, specific absorbance of conjugated dienes (SA), and browning index (BI). In contrast, for the collector fraction, these conditions were met for aw, SA, and the d50 of the reconstituted emulsion (LD).

For the chamber fraction, the yield was well described by the model, with an R^2^ close to 0.9, indicating very good predictive accuracy. Tin was the most influential variable, appearing in both linear (negative) and quadratic terms, while FR had only an inverse linear effect. The experimental results indicate that increasing FR led to lower chamber yields, whereas the highest yields were obtained at the highest Tin value, which is described in the equation with the positive quadratic term.

The aw was mainly affected by the FR, showing a negative linear and a positive quadratic effect, meaning moderate increases in FR reduced aw, but extreme values raised it again. Tin had only a minor negative linear influence, slightly lowering aw, while its positive interaction with the FR suggests combined conditions can increase aw. This model also showed a relatively high explanatory power (R^2^ > 0.8). Similarly, the SA was well described by the model (R^2^ > 0.8). In this case, Tin was the most relevant factor, appearing as a linear, quadratic, and interactive term. This aligns with the experimental results, where powders collected in the chamber showed higher oxidation levels at higher temperatures, likely due to the longer residence time under heat. Finally, the model also adequately described the BI of the chamber powders, with R^2^ values above 0.8. The BI was mainly affected by Tin, as higher temperatures promoted more intense browning, most likely due to Maillard reactions. An inverse effect of the FR was also detected, which is consistent with the previously discussed results: a higher FR shortened the residence time of the powders in the chamber, reducing browning intensity.

Regarding the collector, the aw of powder showed an inverse linear effect of both Tin and FR, as well as a significant positive Tin × FR interaction. This model had an R^2^ above 0.7, indicating a relatively good predictive ability. The SA model also showed an R^2^ above 0.7 and highlighted the strong influence of both Tin and the FR, although the FR appeared to have a more pronounced effect. The positive coefficients for the linear terms suggest that increasing either Tin or the FR leads to higher SA values, indicating greater oil oxidation. However, the negative quadratic coefficient implies that this effect is not strictly linear; beyond a certain point, further increases in Tin or the FR could attenuate or even reverse the trend. The droplet size (d50) of the reconstituted emulsion from the collector powder was well described by the model (R^2^ > 0.8), indicating good predictive ability. Among the variables, the FR had the strongest influence, appearing as a significant linear and quadratic term, as well as in the Tin × FR interaction. This suggests that both very low and very high feed rates tend to produce larger droplets, while an intermediate FR minimises droplet size. Tin also played an important role, showing a negative linear effect, indicating that increasing inlet temperature reduces droplet size. Still, this trend was moderated by a small positive quadratic effect, suggesting a slight curvature at extreme temperatures. Together, these results indicate a complex, non-linear response where the combined effects of Tin and the FR determine the final droplet size of the reconstituted emulsion.

The total yield was also successfully modelled, showing good agreement with the experimental data (R^2^ > 0.8). The response exhibited a direct linear effect of Tin. Conversely, the FR showed a linear inverse impact, indicating that higher feed rates slightly reduced the total yield.

It is worth noting that not all parameters could be modelled with the same accuracy for the collector and chamber fractions because their behaviours were inherently different. For example, the yield remained nearly constant for the collector, while the drying conditions strongly influenced it in the chamber. Likewise, the BI was more affected in the chamber fraction because the powders were exposed to high temperatures for longer periods, enhancing browning reactions.

These clear differences highlight the importance of characterising the powders from the collector and the chamber separately. Each fraction undergoes distinct drying dynamics and thermal exposure, leading to different functional and physicochemical properties.

Figure S1 shows the plots of standardised residuals versus the calculated values for each response variable. In all cases, the residuals were randomly scattered around zero, indicating no evident systematic patterns or model misspecification. Most points were within the ±2 range, with only a few approaching ±3, suggesting the absence of strong outliers or influential observations. The dispersion of residuals appeared relatively constant across the predicted range.

### 3.5. Multifactorial Optimisation

Optimisation was carried out only including the parameters significantly affected by the drying variables and well described by the regression model (R^2^ > 0.5). Three separate optimisations were carried out.

First, the drying conditions were identified to optimise the chamber powder characteristics, with the objective of maximising the yield, which was strongly affected by the drying conditions, while simultaneously minimising aw, SA, and the browning index (BI).

Similarly, the Tin and FR conditions were identified to optimise the collector powder. Since its yield remained very stable regardless of the conditions, the objective function focused on minimising aw, the SA, and the droplet size of the reconstituted emulsion. This approach aimed to obtain a dry powder with low oxidation levels and good emulsion stability.

Finally, a global optimisation was proposed, considering the most relevant characteristics of both powders. In this case, the total yield was maximised while minimising the aw of the collector powder. This criterion was chosen because the chamber powder was inherently drier due to the higher temperatures in the drying chamber; indeed, from 145 °C onward, the chamber powder consistently showed lower aw values than the collector, which was more sensitive to this parameter. The SA and BI of the chamber powders were minimised to reduce oxidation and browning. The SA of the collector powder was not included to avoid duplicating this parameter. Moreover, the highest levels of oil oxidation were observed in the chamber powder, particularly at the highest temperatures (155 °C and 159 °C), indicating that this fraction was more sensitive to oxidative damage.

The desirability functions were defined for each parameter, assigning the same relative importance to all in the overall desirability function.

**Figure 16:**
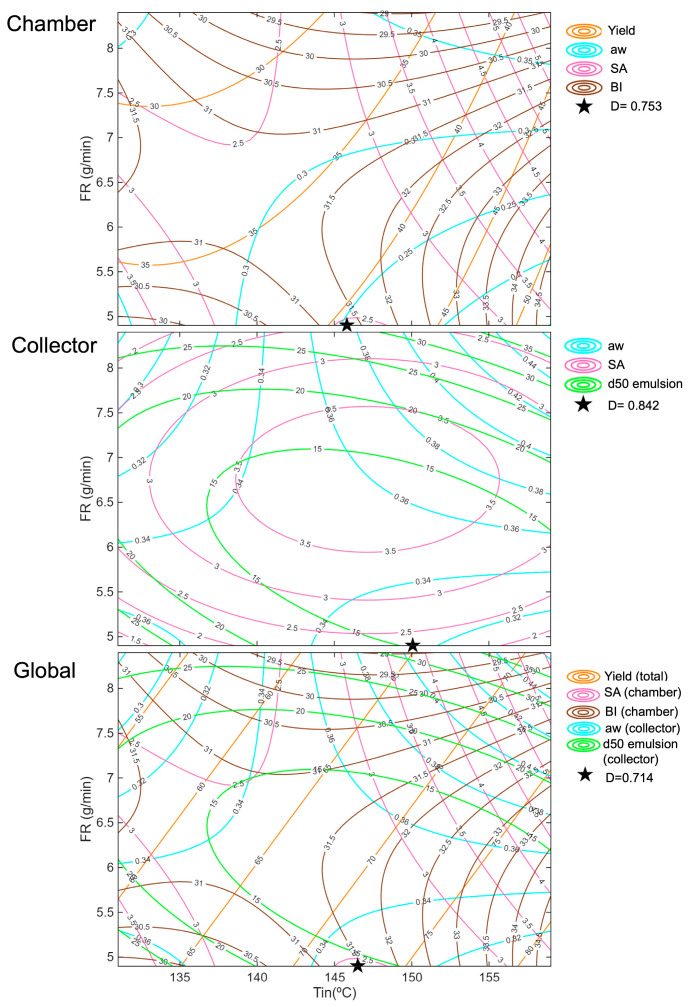
Contour plots showing the optimisation results for the chamber (**top**), collector (**middle**), and global (**bottom**) responses. Each plot represents the simultaneous behaviour of the variables considered in the optimisation: yield, water activity (aw), specific absorbance of conjugated dienes (SA), browning index (BI), and emulsion droplet size (d50). The black star marks the optimal operating conditions with the corresponding overall desirability (D).

The results of the optimisation, including the overall desirability values for each case, are presented in Table 4. Moreover, Figure 16 illustrates the position of the overall desirability in the studied region for each optimisation, along with all the parameters considered in the optimisation process. The overall desirability values obtained for the optimised conditions were 0.753 for the chamber, 0.842 for the collector, and 0.714 for the global optimisation. These values indicate that the collector conditions achieved the best overall compromise among the targeted responses, while the chamber and global optimisations were still satisfactory.

The optimisation for the chamber represents a balanced compromise between yield, BI, SA, and aw because it selects conditions where none of the responses reach their extremes (intermediate Tin of 146 °C and the lowest FR of 4.9 g/min). Very high temperatures increased yield but also intensified oxidation (SA), while low temperatures reduced oxidation but resulted in poor yield. Similarly, the effect of FR was non-linear: in some cases, increasing FR reduced browning due to shorter residence times, but in others, it increased aw and even worsened oxidation. The chosen optimum at moderate Tin and low FR achieves a middle ground; yield is enhanced compared to a very high FR, while oxidation and browning are kept lower than at the highest temperatures, and aw remains stable. This reflects the trade-off captured by the model, prioritising overall product quality without excessively sacrificing any single parameter.

For the collector powder, optimisation focused on minimising aw, SA, and the droplet size of the reconstituted emulsion. The optimum corresponded to a low FR (4.9 g/min) combined with an intermediate Tin (150 °C, similar conditions to the chamber with slightly higher Tin), which represented the best compromise among the three parameters. According to the model and experimental results, an excessively high FR increased aw and, in some cases, promoted oil oxidation (SA) due to inefficient drying and higher residual moisture. Similarly, a high FR produced larger droplets, while an intermediate-to-low FR minimised d50, improving emulsion stability. Regarding temperature, higher Tin generally reduced droplet size due to faster crust formation but also increased oxidation beyond a certain point. Conversely, too low Tin risked incomplete drying and higher aw. Thus, the optimisation favoured a moderate Tin, which balanced proper drying without excessive thermal damage, together with a lower FR.

The global optimisation closely matched the individual optimisations, aligning perfectly with the chamber fraction. This is because the chamber responses, yield, oxidation (SA), and browning (BI), were more sensitive to drying conditions, especially temperature, making them dominant in the global solution. While the collector focused mainly on minimising aw, SA, and droplet size, its influence was negligible. Thus, the global optimum reflects a compromise but primarily favours the chamber’s critical parameters while keeping acceptable conditions for the collector.

Similar trends have been reported in the literature, supporting the low feed rates identified in our optimisation. For example, Himmetagaoglu and Erbay [55], who spray-dried high-fat dairy emulsions to produce cream powders, observed that higher feed rates produced powders with lower quality, higher surface fat, and wettability. Likewise, Yadav et al. [56], working on probiotic microencapsulation via spray drying, showed that increasing the feed rate reduced yield and worsened aw due to insufficient drying time inside the chamber. These findings align with our results, where an excessively high FR increased aw, occasionally promoted oxidation, and produced larger droplets. At the same time, a low FR ensured better drying, improved emulsion stability, and minimised quality losses.

To validate the model predictions, experiments were carried out under the optimal spray-drying conditions determined for each powder fraction. As shown in Table 4, the experimental values closely matched the predicted responses across most of the evaluated parameters. For the chamber fraction, yield, SA, and BI exhibited good agreement with the model, confirming the reliability of the predicted optimum conditions. The experimental value for aw was slightly larger but still acceptably similar to the predicted one. Similarly, for the collector powder, the predicted and experimental values of aw, SA, and d50 of the reconstituted emulsion fell within acceptable deviation margins. In the case of the global optimisation, the experimentally measured responses of d50 were slightly higher than predicted but still within reasonable agreement. These findings support the validity of the models and confirm their applicability for guiding process optimisation under different recovery positions, reinforcing the robustness of the methodological approach used.

## 4. Conclusions

This study evaluated the spray drying of double-layer emulsions stabilised with a sustainable wall material composed of orange residue flour (ORF), soy protein, and maltodextrin, analysing the effect of inlet temperature (Tin) and feed rate (FR) on yield, encapsulation efficiency, oil oxidation, water activity, particle size, and microstructure. Experimental data were successfully modelled using response surface methodology (RSM), which allowed prediction of powder characteristics and identification of optimal operating conditions for each fraction. Chamber powders were strongly influenced by Tin, with higher temperatures (155–159 °C) increasing yield (up to 48% dm) but also promoting oil oxidation, while collector powders showed more stable yields (≈30% dm) but greater sensitivity of aw. Microstructural analysis of the reconstituted emulsions revealed that most of the insoluble ORF particles remained in the chamber fraction, influencing particle size, colour, and encapsulation efficiency.

RSM optimisation indicated that the best compromise for chamber powders was achieved at a moderate Tin (≈146 °C) combined with the lowest FR (4.9 g/min), balancing yield and stability. For collector powders, the optimal conditions were Tin = 150 °C with the same low FR. The modelling approach accurately predicted experimental values and provides a practical tool for adjusting drying conditions according to the desired product fraction.

The results are directly applicable to the development of powdered functional ingredients enriched with vegetable oils or lipid-soluble bioactives, such as clean-label additives, fortified beverages, or instant mixes, where oxidative stability is critical. The use of ORF as a wall material offers a sustainable alternative that promotes the valorisation of agro-industrial residues while maintaining high encapsulation performance.

This work was conducted at laboratory scale, and shelf life or functional behaviour in real food matrices was not assessed. Future research should address scale-up to pilot and industrial spray dryers, evaluate performance during storage and processing in target food systems, and explore the applicability of this approach to other oil-in-water or multilayer emulsions.

## Figures and Tables

**Table 1:** Conditions of all the experiments performed.

Run	Tin (°C)	% FR	FR (g/min)	Tout (°C)
1	135	25	8.0	88–92
2	135	15	5.5	92–95
3	145	20	6.8	90–97
4	155	15	5.5	101–102
5	155	25	8.0	96–99
6	145	20	6.8	96–98
7	145	20	6.8	90–94
8	145	20	6.8	94–96
9	159	20	6.8	108–109
10	145	13	4.9	92–95
11	131	20	6.8	88–90
12	145	27	8.4	94–96

**Table 2:** Results of the three-way ANOVA, considering inlet temperature (Tin), feed rate (FR), and powder collection position as factors.

	Yield			EE				MC				aw				SA			d50 Emulsion (LD)			
Factor	F-Value		***p*** **^#^**	F-Value			***p*** **^#^**	F-Value		***p*** **^#^**		F-Value		***p*** **^#^**		F-Value	***p*** **^#^**		F-Value		***p*** **^#^**	
Tin	19.7		***	3.0			*	27.0		***		6.6		***		75.6	***		2.5		ns	
FR	9.5		***	0.4			ns	12.4		***		83.8		***		50.0	***		5.0		**	
Position	105.7		***	488.4			***	32.6		***		348.2		***		1.4	ns		606.2		***	
Tin/FR	0.0		ns	3.7			ns	20.8		***		201.0		***		41.1	***		2.7		ns	
Tin/Position	9.5		***	2.4			ns	8.4		***		33.9		***		105.2	***		3.8		**	
FR/Position	7.2		***	4.7			**	3.8		*		14.4		***		34.3	***		2.3		ns	
Tin/FR/Position	1.1		ns	0.6			ns	3.8		ns		1.7		ns		68.7	***		0.4		ns	
		**d50 Emulsion (IA)**				**Sphericity (Emulsion)**					**d50 Powder**				**Sphericity (Powder)**					**BI**		
**Factor**		**F-Value**			***p*** **^#^**	**F-Value**			***p*** **^#^**		**F-Value**		***p*** **^#^**		**F-Value**			***p*** **^#^**		**F-Value**		***p*** **^#^**
Tin		3.5			*	1.2			ns		57.2		***		6.0			***		4.6		**
FR		4.6			**	2.8			*		0.2		ns		2.3			ns		1.8		ns
Position		0.7			ns	468.9			***		3774.4		***		5.1			*		3900.6		***
Tin/FR		0.3			ns	0.0			ns		0.9		ns		1.0			ns		7.9		*
Tin/Position		3.8			**	1.5			ns		14.2		***		2.3			ns		7.6		***
FR/Position		9.3			***	4.6			**		1.0		ns		5.1			**		7.7		***
Tin/FR/Position		10.4			**	0.0			ns		0.1		ns		0.4			ns		2.1		ns

**^#^** Significance codes: ***: *p* < 0.001; **: *p* < 0.01; *: *p* < 0.05; ns: no significant.

**Table 3:** Estimated regression coefficients and goodness-of-fit statistics (R^2^ and adjusted R^2^).

	Response	Intercept	Tin	FR	Tin^2^	FR^2^	Tin/FR	R^2^	Adjusted R^2^
Chamber	Yield	463.41	−6.203	−2.82	0.0233	0	0	0.896	0.857
	aw	4.02	−0.0231	−0.6	0	0.0123	0.0032	0.816	0.711
	SA	160.13	−1.9303	−6.769	0.0058	0	0.0476	0.814	0.708
	BI	190.65	−2.2053	−0.6239	0.0078	0	0	0.556	0.390
Collector	aw	2.495	−0.0153	−0.3667	0	0	0.0026	0.782	0.700
	SA	−101.64	1.1636	5.866	−0.003954	−0.4341	0	0.718	0.557
	d50 emulsion (LD)	963.1	−9.6635	−77.527	0.027	3.1614	0.268	0.858	0.739
Global	Yield	−13.44	0.688	−2.95	0	0	0	0.840	0.805

**Table 4:** Predicted and experimental responses at the optimal spray drying conditions for chamber, collector, and global optimisation.

	Optimal Conditions				
Position	Tin (°C)	FR (g/min)		Predicted Response	Experimental Response
Chamber	146	4.9	Yield (% dm)	40.7	42.7 ± 2.6
			aw	0.247	0.278 ± 0.001
			SA	2.48	2.68 ± 0.19
			BI	31.3	31.3 ± 0.4
Collector	150	4.9	aw	0.324	0.336 ± 0.007
			SA	2.30	2.48 ± 0.15
			d50 emulsion (LD) (µm)	14.7	15.5 ± 5.5
Global	146	4.9	Yield (% dm)	72.9	74.9 ± 4.0
			SA (chamber)	2.48	2.68 ± 0.19
			BI (chamber)	31.4	31.3 ± 0.4
			aw (collector)	0.332	0.341 ± 0.001
			d50 emulsion (LD) (µm)	16.0	18.0 ± 2.8

## Data Availability

The dataset is available on request from the authors.

## Supplementary Materials

The following supporting information can be downloaded at: https://www.mdpi.com/article/10.3390/foods14162919/s1, Figure S1: Standard residuals vs. calculated values of the model responses for the chamber, collector and global.

## Abbreviations

The following abbreviations are used in this manuscript:

aw	Water activity
BI	Browning index
d50	Median particle diameter (50th percentile)
EE	Encapsulation efficiency
FR	Feed rate
IA	Image analysis
LD	Laser diffraction
MC	Moisture content
ORF	Orange residue flour
PSD	Particle size distribution
RSM	Response surface methodology
SA	Specific absorbance of conjugated dienes (234 nm)
SEM	Scanning electron microscopy
Tin	Inlet temperature
Tout	Outlet temperature

## References

[1] Zahran H.A., Catalkaya G., Yenipazar H., Capanoglu E., Şahin-Yeşilçubuk N. (2023). Determination of the Optimum Conditions for Emulsification and Encapsulation of Echium Oil by Response Surface Methodology. ACS Omega.

[2] Comunian T.A., Gomez-Estaca J., Ferro-Furtado R., Conceição G.J.A., Moraes I.C.F., de Castro I.A., Favaro-Trindade C.S. (2016). Effect of Different Polysaccharides and Crosslinkers on Echium Oil Microcapsules. Carbohydr. Polym..

[3] Fernandes B., Oliveira M.C., Marques A.C., dos Santos R.G., Serrano C. (2024). Microencapsulation of Essential Oils and Oleoresins: Applications in Food Products. Foods.

[4] Díaz-Montes E. (2023). Wall Materials for Encapsulating Bioactive Compounds via Spray-Drying: A Review. Polymers.

[5] Shi Z., Chen Z., Meng Z. (2023). Study on Oil Body Emulsion Gels Stabilized by Composited Polysaccharides through Microgel Particles Compaction and Natural Gelation. Food Hydrocoll..

[6] Faustino M., Veiga M., Sousa P., Costa E.M., Silva S., Pintado M. (2019). Agro-Food Byproducts as a New Source of Natural Food Additives. Molecules.

[7] Burgos-Díaz C., Leal-Calderon F., Mosi-Roa Y., Chacón-Fuentes M., Garrido-Miranda K., Opazo-Navarrete M., Quiroz A., Bustamante M. (2024). Enhancing the Retention and Oxidative Stability of Volatile Flavors: A Novel Approach Utilizing O/W Pickering Emulsions Based on Agri-Food Byproducts and Spray-Drying. Foods.

[8] Dalmau E., Osselló C., Eim V., Ratti C., Simal S. (2020). Ultrasound-Assisted Aqueous Extraction of Biocompounds from Orange Byproduct: Experimental Kinetics and Modelling. Antioxidants.

[9] Umaña M.M., Dalmau M.E., Eim V.S., Femenia A., Rosselló C. (2019). Effects of Acoustic Power and Ph on Pectins-enriched Extracts Obtained from Citrus By-products. Model. Extr. Process. J. Sci. Food Agric..

[10] FAOSTAT. https://www.fao.org/faostat/es/#data/QCL.

[11] Panwar D., Panesar P.S., Chopra H.K. (2021). Recent Trends on the Valorization Strategies for the Management of Citrus By-Products. Food Rev. Int..

[12] Panwar D., Saini A., Panesar P.S., Chopra H.K. (2021). Unraveling the Scientific Perspectives of Citrus By-Products Utilization: Progress towards Circular Economy. Trends Food Sci. Technol..

[13] Umaña M., Llull L., Bon J., Eim V.S., Simal S. (2022). Artificial Neural Networks to Optimize Oil-in-Water Emulsion Stability with Orange By-Products. Foods.

[14] Willats W.G.T., Knox J.P., Mikkelsen J.D. (2006). Pectin: New Insights into an Old Polymer Are Starting to Gel. Trends Food Sci. Technol..

[15] Maravić N., Šereš Z., Nikolić I., Dokić P., Kertész S., Dokić L. (2019). Emulsion Stabilizing Capacity of Sugar Beet Fibers Compared to Sugar Beet Pectin and Octenyl Succinate Modified Maltodextrin in the Production of O/W Emulsions: Individual and Combined Impact. LWT.

[16] Neckebroeck B., Verkempinck S.H., Van Audenhove J., Bernaerts T., de Wilde d’Estmael H., Hendrickx M.E., Van Loey A.M. (2020). Structural and Emulsion Stabilizing Properties of Pectin Rich Extracts Obtained from Different Botanical Sources. Food Res. Int..

[17] Rodrigues F.J., Cedran M.F., Bicas J.L., Sato H.H. (2020). Encapsulated Probiotic Cells: Relevant Techniques, Natural Sources as Encapsulating Materials and Food Applications—A Narrative Review. Food Res. Int..

[18] Moser P., Locali-Pereira A.R., Nicoletti V.R. (2024). Optimization of Spray Drying Process of Buriti Oil-Loaded Bilayer Emulsions. Colloids Surf. A Physicochem. Eng. Asp..

[19] Locali A.R., Cattelan M. (2019). of pink pepper essential oil: P. of spray-dried pectin/SPI double-layer versus S. single-layer stabilized emulsions G.; Nicoletti, V.R. Microencapsulation of Pink Pepper Essential Oil: Properties of Spray-Dried Pectin/SPI Double-Layer versus SPI Single-Layer Stabilized Emulsions. Colloids Surf. A Physicochem. Eng. Asp..

[20] Moser P., Nicoletti V.R., Drusch S., Brückner-Gühmann M. (2020). Functional Properties of Chickpea Protein-Pectin Interfacial Complex in Buriti Oil Emulsions and Spray Dried Microcapsules. Food Hydrocoll..

[21] Zhang T., Luo Y., Wang M., Chen F., Liu J., Meng K., Zhao H. (2020). Double-Layered Microcapsules Significantly Improve the Long-Term Effectiveness of Essential Oil. Polymers.

[22] Nascimento A.P.S., Carvalho A.J.D.B.A., Lima M.D.S., Barros S.L., Ribeiro S., Pasqualli M., Lisboa H.M., Barros A.N. (2023). Enhancing Antioxidant Retention through Varied Wall Material Combinations in Grape Spray Drying and Storage. Antioxidants.

[23] Čulina P., Zorić Z., Garofulić I.E., Repajić M., Dragović-Uzelac V., Pedisić S. (2023). Optimization of the Spray-Drying Encapsulation of Sea Buckthorn Berry Oil. Foods.

[24] Dang Y.T., Tran H., Kha T.C. (2024). Encapsulation of W/O/W Acerola Emulsion by Spray Drying: Optimization, Release Kinetics, and Storage Stability. Foods.

[25] Donz E., Boiron P., Courthaudon J.-L. (2014). Characterization of Industrial Dried Whey Emulsions at Different Stages of Spray-Drying. J. Food Eng..

[26] Hernández-Sanchez M.D.R.H., Cuvelier M.E., Turchiuli C. (2015). Design of Liquid Emulsions to Structure Spray Dried Particles. J. Food Eng..

[27] Pieczykolan E., Kurek M.A. (2019). Use of Guar Gum, Gum Arabic, Pectin, Beta-Glucan and Inulin for Microencapsulation of Anthocyanins from Chokeberry. Int. J. Biol. Macromol..

[28] McIlvaine T.C. (1921). A buffer solution for colorimetric comparison. J. Biol. Chem..

[29] Umaña M., Wawrzyniak P., Rosselló C., Llavata B., Simal S. (2021). Evaluation of the Addition of Artichoke By-Products to O/W Emulsions for Oil Microencapsulation by Spray Drying. LWT.

[30] Umaña M., Turchiuli C., Eim V., Rosselló C., Simal S. (2021). Stabilization of Oil-in-Water Emulsions with a Mushroom (*Agaricus bisporus)* by-Product. J. Food Eng..

[31] Turchiuli C., Munguia M.T.J., Sanchez M.H., Ferre H.C., Dumoulin E. (2014). Use of Different Supports for Oil Encapsulation in Powder by Spray Drying. Powder Technol..

[32] Turchiuli C., Lemarié N., Cuvelier M.E., Dumoulin E. (2013). Production of Fine Emulsions at Pilot Scale for Oil Compounds Encapsulation. J. Food Eng..

[33] Talón E., Lampi A.-M., Vargas M., Chiralt A., Jouppila K., González-Martínez C. (2019). Encapsulation of Eugenol by Spray-Drying Using Whey Protein Isolate or Lecithin: Release Kinetics, Antioxidant and Antimicrobial Properties. Food Chem..

[34] Umaña M., Bon J., Wawrzyniak P., Eim V., Simal S. Effect of the Spray Drying Conditions in the Microencapsulation of Oil Using Orange By-Products. Proceedings of the Effect of the Spray Drying Conditions in the Microencapsulation.

[35] Mousavi Kalajahi S.E., Ghandiha S. (2022). Optimization of Spray Drying Parameters for Encapsulation of Nettle (*Urtica dioica* L.) Extract. LWT.

[36] GEA-Niro Analytical Methods for Dry Milk Products. https://www.gea.com/en/products/analytical-methods-dry-milk-products.jsp.

[37] Kim E.H., Chen X.D., Pearce D. (2005). Melting Characteristics of Fat Present on the Surface of Industrial Spray-Dried Dairy Powders. Colloids Surf. B Biointerfaces.

[38] AOAC (2000). Method 934.06 Moisture in Dried Fruits. AOAC Int..

[39] Sánchez M.D.R.H., Cuvelier M.E., Turchiuli C. (2016). Effect of α-Tocopherol on Oxidative Stability of Oil during Spray Drying and Storage of Dried Emulsions. Food Res. Int..

[40] Buera M.P., Petriella C., Lozano R.D. (1985). Definition of Colour in the Non-Enzymatic Browning. Die Farbe.

[41] Bezerra M.A., Ferreira S.L.C., Novaes C.G., Dos Santos A.M.P., Valasques G.S., Da Mata Cerqueira U.M.F., Dos Santos Alves J.P. (2019). Simultaneous Optimization of Multiple Responses and Its Application in Analytical Chemistry—A Review. Talanta.

[42] Einhorn-Stoll U. (2018). Pectin-Water Interactions in Foods–From Powder to Gel. Food Hydrocoll..

[43] Garau M.C., Simal S., Rosselló C., Femenia A. (2007). Effect of Air-Drying Temperature on Physico-Chemical Properties of Dietary Fibre and Antioxidant Capacity of Orange (*Citrus aurantium* v. Canoneta) By-Products. Food Chem..

[44] Umaña M., Turchiuli C., Rosselló C., Simal S. (2021). Addition of a Mushroom By-Product in Oil-in-Water Emulsions for the Microencapsulation of Sunflower Oil by Spray Drying. Food Chem..

[45] Quintero-Gamero G., Sánchez-Garzón F.S., Rodríguez-Cortina A., Hernández-Carrión M., Nerio L.S. (2025). Spray Drying of Buriti Oil-in-Water Emulsions as Potential Systems for the Delivery of Bioactive Compounds. Appl. Food Res..

[46] Gonçalves A., Estevinho B.N., Rocha F. (2022). Spray-Drying of Oil-in-Water Emulsions for Encapsulation of Retinoic Acid: Polysaccharide- and Protein-Based Microparticles Characterization and Controlled Release Studies. Food Hydrocoll..

[47] Domínguez R., Pateiro M., Gagaoua M., Barba F.J., Zhang W., Lorenzo J.M. (2019). A Comprehensive Review on Lipid Oxidation in Meat and Meat Products. Antioxidants.

[48] Javed A., Imran M., Ahmad N., Hussain A.I. (2018). Fatty Acids Characterization and Oxidative Stability of Spray Dried Designer Egg Powder. Lipids Health Dis..

[49] Huang H., Hao S., Li L., Yang X., Cen J., Lin W., Wei Y. (2014). Influence of Emulsion Composition and Spray-Drying Conditions on Microencapsulation of Tilapia Oil. J. Food Sci. Technol..

[50] Höhne S., Gaukel V. (2024). Impact of the Drying Rate on Product Properties of Spray Dried Emulsions to Enable a Targeted Product Design. Dry. Technol..

[51] Peng Q., Meng Z., Luo Z., Duan H., Ramaswamy H.S., Wang C. (2023). Effect of Emulsion Particle Size on the Encapsulation Behavior and Oxidative Stability of Spray Microencapsulated Sweet Orange Oil (*Citrus aurantium* var. Dulcis). Foods.

[52] Jiang W., Wang J., Yang Y., Bu Y. (2024). A Review of Microsphere Super-Resolution Imaging Techniques. Sensors.

[53] Laser Diffraction (LD): Particle Size Analyzers: Microtrac. https://www.microtrac.com/products/particle-size-shape-analysis/laser-diffraction/.

[54] Çalışkan Koç G., Nur Dirim S. (2017). Spray Drying of Spinach Juice: Characterization, Chemical Composition, and Storage. J. Food Sci..

[55] Himmetagaoglu A.B., Erbay Z. (2019). Effects of Spray Drying Process Conditions on the Quality Properties of Microencapsulated Cream Powder. Int. Dairy J..

[56] Yadav S., Mishra S. (2024). Optimization of Spray-Drying Process for the Microencapsulation of L. Plantarum (MCC 2974) in Ultrasound Hydrated Finger Millet Milk. Food Sci. Biotechnol..

