# Supplementary material for: Spray Drying of Double-Layer Emulsion Stabilised with an Orange Residue: Effect of Process Parameters and Collection Position
Source: Foods. 2025 Aug 21;14(16):2919. doi: 10.3390/foods14162919 (PMC12385256; doi:10.3390/foods14162919)
Supplement: Supplementary file 1 [file foods-14-02919-s001.zip › foods-3816575-supplementary.pdf]

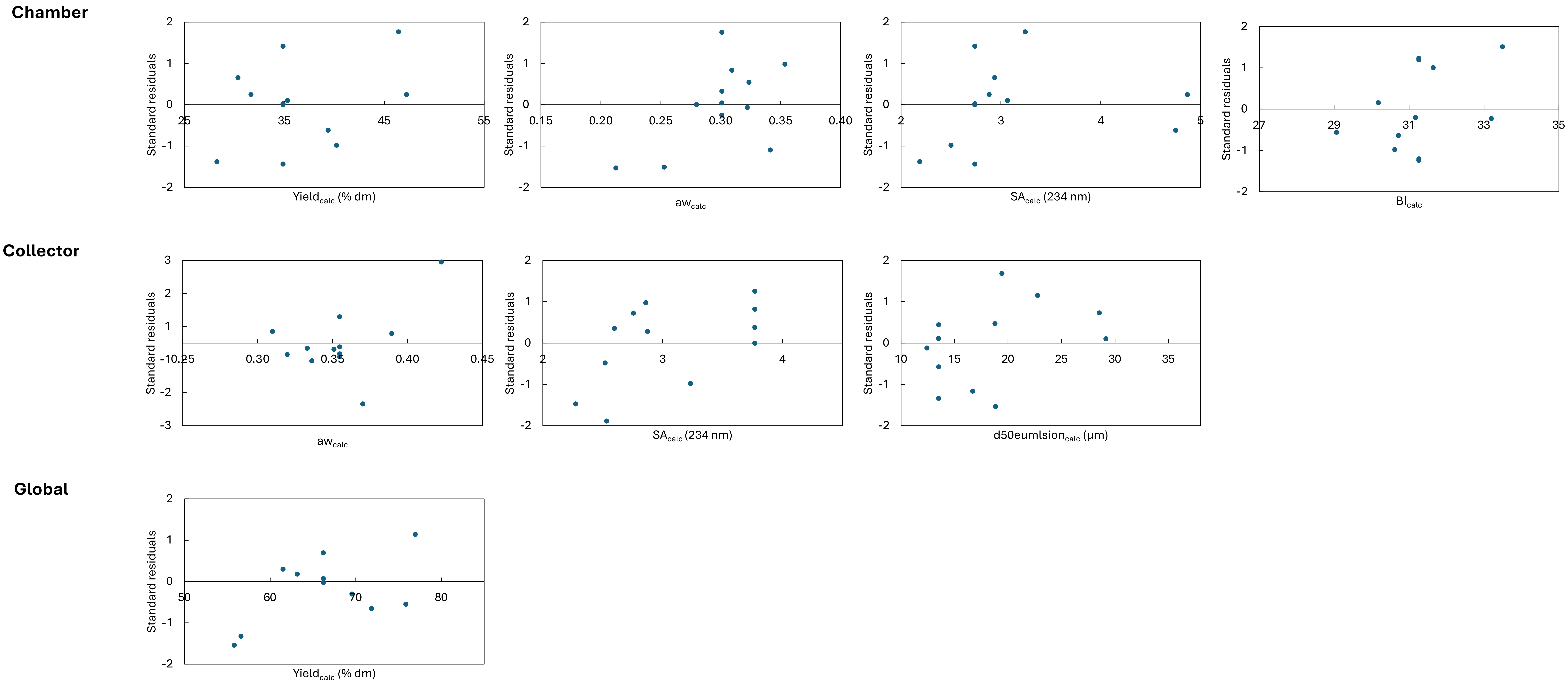

Figure S1. Standard residuals vs calculated values of the model responses for the chamber, collector and global.
